# Fibroblast growth factor (FGF), FGF receptor (FGFR), and cyclin D1 (*CCND1*) DNA methylation in head and neck squamous cell carcinomas is associated with transcriptional activity, gene amplification, human papillomavirus (HPV) status, and sensitivity to tyrosine kinase inhibitors

**DOI:** 10.1186/s13148-021-01212-4

**Published:** 2021-12-21

**Authors:** Yilin Bao, Jennis Gabrielpillai, Jörn Dietrich, Romina Zarbl, Sebastian Strieth, Friederike Schröck, Dimo Dietrich

**Affiliations:** 1 Department of Otorhinolaryngology, Head and Neck Surgery, University Medical Center Bonn (UKB), Sigmund-Freud-Str. 25, 53105 Bonn, Germany; 2 Department of Otolaryngology, Head and Neck Surgery, The Affiliated Hospital of Southwest Medical University, Luzhou, China; 3 Department of Psychiatry and Psychotherapy, University Hospital Bonn, Bonn, Germany

**Keywords:** Fibroblast growth factor receptor (FGFR), Fibroblast growth factor (FGF), Cyclin D1 (*CCND1*), DNA methylation, Head and neck squamous cell carcinoma (HNSCC), Tyrosine kinase inhibitor (TKI), Human papillomavirus (HPV), Predictive biomarker

## Abstract

**Background:**

Dysregulation of fibroblast growth factor receptor (FGFR*)* signaling pathway has been observed in head and neck squamous cell carcinoma (HNSCC) and is a promising therapeutic target for selective tyrosine kinase inhibitors (TKIs). Potential predictive biomarkers for response to FGFR-targeted therapies are urgently needed. Understanding the epigenetic regulation of FGF pathway related genes, i.e. FGFRs, FGFs, and *CCND1*, could enlighten the way towards biomarker-selected FGFR-targeted therapies.

**Methods:**

We performed DNA methylation analysis of the encoding genes *FGFR1, FGFR2*, *FGFR3, FGFR4, FGF1-14*, *FGF16-23*, and *CCND1* at single CpG site resolution (840 CpG sites) employing The Cancer Genome Research Atlas (TCGA) HNSCC cohort comprising *N* = 530 tumor tissue and *N* = 50 normal adjacent tissue samples. We correlated DNA methylation to mRNA expression with regard to human papilloma virus (HPV) and gene amplification status. Moreover, we investigated the correlation of methylation with sensitivity to the selective FGFR inhibitors PD 173074 and AZD4547 in *N* = 40 HPV(−) HNSCC cell lines.

**Results:**

We found sequence-contextually nuanced CpG methylation patterns in concordance with epigenetically regulated genes. High methylation levels were predominantly found in the promoter flank and gene body region, while low methylation levels were present in the central promoter region for most of the analyzed CpG sites. FGFRs, FGFs, and *CCND1* methylation differed significantly between tumor and normal adjacent tissue and was associated with HPV and gene amplification status. *CCND1* promoter methylation correlated with *CCND1* amplification*.* For most of the analyzed CpG sites, methylation levels correlated to mRNA expression in tumor tissue. Furthermore, we found significant correlations of DNA methylation of specific CpG sites with response to the FGFR1/3–selective inhibitors PD 173074 and AZD4547, predominantly within the transcription start site of *CCND1*.

**Conclusions:**

Our results suggest an epigenetic regulation of *CCND1*, FGFRs, and FGFs via DNA methylation in HNSCC and warrants further investigation of DNA methylation as a potential predictive biomarker for response to selective FGFR inhibitors in clinical trials.

**Supplementary Information:**

The online version contains supplementary material available at 10.1186/s13148-021-01212-4.

## Background

90% of all head and neck cancers are histologically defined as head and neck squamous cell carcinoma (HNSCC), which is the eighth leading cancer by incidence worldwide [1]. Common external risk factors known to contribute to the development of HNSCC are tobacco and alcohol abuse, accounting for approximately 75% of HNSCCs [2]. Another subset of HNSCCs, especially oropharyngeal carcinomas, are caused by an infection with the human papillomavirus (HPV). This subset of HNSCCs mostly occurs in tissue of the tonsils. HPV DNA is found in 45–67% of these cases [3].

Most individuals with HNSCC are diagnosed at an advanced stage [4]. Treatment with primary surgery or radiotherapy improves the cure rate of early-stage HNSCC patients with a 5-year overall survival (OS) of 70–90%. However, the prognosis at advanced stages is still poor [5], and differs significantly on the basis of tumor site, HPV infection status, and overall stage. According to Pulte et al., the 5-year relative survival rate decreased significantly for patients with metastatic disease, with survival rates of 41.5%, 32.5%, and 29.5% for tonsillar carcinoma, carcinoma of the tongue, and oral cavity malignancies, respectively [6].

To date, it is well acknowledged that alcohol-/smoking-related and HPV-induced cancers represent distinct tumor entities with different molecular and clinical features. Next to distinct outcome and prognosis [7], diverging genomic profiles contribute to tumor heterogeneity, also within subgroups of HPV-negative (HPV(−)) HNSCCs [8]. This suggests a completely different tumor biology, thus bringing up the need for specific individual therapeutic strategies, including predictive and prognostic biomarkers supporting treatment decisions. Genetic and epigenetic profiles of HNSCCs are currently in the focus of biomarker research.

Amongst different therapeutic concepts, targeted therapies have changed the treatment landscape of cancer disease magnificently over the past years. Since the 1980s, receptor tyrosine kinases (RTKs) were recognized to have a critical role in the development and progression of malignant diseases. Consequently, a milestone in cancer treatment was the introduction of tyrosine kinase inhibitors (TKIs) as new targeted therapy strategies in treatment of various malignancies [9].

According to the comprehensive integrative genomic analysis of The Cancer Genome Atlas (TCGA), the most frequently genetically altered RTKs in HNSCC are epidermal growth factor receptor (*EGFR*) / epidermal growth factor receptor 2 (*ERBB2*), and fibroblast growth factor receptors 1/3 (*FGFR1*/*3*): 15% *EGFR*, 4% *ERBB2*, 10% *FGFR1*, and 2% *FGFR3* alterations in HPV(−) HNSCC; 11% *FGFR3* and 3% *ERBB2* mutations in HPV-positive (HPV(+)) HNSCC [8]. While EGFR-targeted therapies employing the monoclonal antibody cetuximab are already included in the standard repertoire of HNSCC treatment, the efficacy of FGFR-inhibition still needs to be determined.

Multiple small molecule inhibitors targeting FGFRs have been developed and tested in studies treating patients suffering from solid tumors including HNSCC. Treatment with the selective FGFR1-4 TKI rogaratinib resulted in only partial responses in two out of ten HNSCC cases in a phase I trial (ClinicalTrials.gov Identifier: NCT01976741). Patients were included based on previously detected FGFR mRNA levels [10]. Several clinical trials are currently underway exploring the response to TKI-inhibitors, such as selective FGFR2 TKI pemigatinib (Incyte Inc., Wilmington, DE, USA; NCT04003623, NCT03822117), FGFR1-4 TKI infigratinib (QED Therapeutics, San Francisco, CA, USA; Inc NCT02706691, NCT01928459), FGFR1-4 inhibitor erdafitinib (Janssen Biotech Inc., Horsham, PA, USA; NCT04083976, NCT03210714), FGFR1-3 inhibitor Debio 1347 (Debiopharm, Lausanne, Switzerland; NCT03834220), and the selective FGFR1-4 inhibitor rogaratinib (Bayer AG, Leverkusen, Germany; NCT02592785). Recently, treatment of locally advanced and unresectable or metastatic urothelial carcinoma carrying at least one *FGFR3* mutation or *FGFR2*/*3* fusion with the selective FGFR-TKI erdafitinib has shown impressive efficacy ultimately leading to the US Food and Drug Administration (FDA) approval [11]. Predictive properties of *FGFR2* fusions/translocations in response to selective FGFR kinase inhibitor were confirmed in a phase II study for the treatment of advanced cholangiocarcinoma (NCT02150967) [12]. *FGFR* alterations, such as gene amplifications, oncogenic fusions or increased ligand expression have shown to cause increased and/or abnormal signaling activity [13–16]. Unfortunately, to date there are no predictive biomarkers for treatment of HNSCC with FGFR inhibitors. Although a number of patients with HNSCC have had benefit from treatment with TKI, it remains widely unclear which subgroup, even if small, should be included in this therapeutic strategy [17].

The FGFR family consists of four members, FGFR1-4, which share high homology, with their sequence identity varying from 56% to 71% [18]. Similar to other RTKs, FGFRs can be stimulated and activated by extracellular signals. The native ligands of FGFRs are fibroblast growth factors (FGFs) [19]. FGFs are classified into six subfamilies: Five paracrine subfamilies and an endocrine subfamily based on sequence homology and developmental characteristics [20]. The five paracrine subfamilies are FGF1 (FGF1 and FGF2), FGF4 (FGF4, FGF5, and FGF6), FGF7 (FGF3, FGF10, FGF7, and FGF22), FGF8 (FGF8, FGF17, and FGF18), and FGF9 (FGF9, FGF16, and FGF20). The FGF19 subfamily (FGF19, FGF21, and FGF23) are endocrine FGFs acting through endocrine secretion [21]. FGF11-FGF14 are not classified into the above six subfamilies. Although they are highly homologous to the other FGF family members, they do not activate FGFRs [22]. According to TCGA data analysis in *N* = 279 HNSCC tumors with previously identified HPV-status, aberrations (amplifications and mutations) of *FGFR1* (10%), *FGFR2* (2%), *FGFR3* (2%), and *CCND1* (31%) was frequently found in HPV(−) tumors. *FGFR3 *(11%, half of them *FGFR3*-*TACC3* fusions) and *CCND1* (3%) aberrations were detected in HPV(+) tumors [8]. Aberrant regulation of the cell cycle is characteristic for all types of cancer. In HNSCC, it is often associated with the overexpression of *CCND1* [23]. Also, *CCND1* co-localizes with *FGF3*, *FGF4*, and *FGF19* at chromosome location 11q13. We therefore included *CCND1* to our list of genes of interest. Co-amplification of those genes is a frequent event in HNSCC [24]. Of note, a particularly high prevalence of *FGF* amplifications can be found in HNSCC, particularly affecting *FGF3* (22.9%), *FGF4* (21.2%), and *FGF19* (22.6%) [24]. A case report describes a complete response to a FGFR Inhibitor with HNSCC harboring *FGF19*, *FGF4*, *FGF23*, and *FGF3* amplifications [25].

In contrast to genetic and genomic alterations, epigenetic features serving as predictive biomarkers for response to TKI are largely unknown. DNA methylation is frequently described as an epigenetic silencing mark [26]. Methylation of cytosine residues in the context of CpG dinucleotide is an important epigenetic mechanism fundamentally contributing to physiological processes, such as transcriptional regulation, cell differentiation, and development, as well as pathological processes influencing carcinogenesis and tumor progression [27, 28]. The clinical utility of predictive DNA methylation biomarkers is well established. DNA promoter methylation of the *MGMT* (O^6^-methylguanine–DNA methyltransferase) DNA repair gene for example is now widely employed as a predictive biomarker in glioblastoma patients’ selection for treatment with the DNA alkylating agent temozolomide [29].

Our present study aims to provide a comprehensive overview of the *FGFR1-4*, *FGF1-14*, *FGF16-23*, and *CCND1* DNA methylation landscape with regard to mRNA expression, *FGFR1* and *CCND1* gene amplification as well as response to FGFR-targeted TKI.

## Materials and methods

### Patients and tumor samples

Molecular data of the HNSCC cohort were obtained from TCGA Research Network (http://cancergenome.nih.gov/) [8]. 530 cancer tissue samples were included (279/530 HNSCC samples with known HPV status, 36/279 HPV(+) samples and 243/279 HPV(−) samples). Among 243 tumor samples with known HPV(−) infection status, 23/243 (9%) of HPV(−) cases harbor *FGFR1* and 76/243 (31%) *CCND1* amplifications. 9/243 (4%) of HPV(−) tumors were *CCND1*/*FGFR1* co-amplified. One HPV(+) case (3%) showed an *CCND1* amplification. No *FGFR1* amplification was present in HPV(+) tumors. 50 normal adjacent tissue (NAT) samples were available [8].

For validation purposes, we further included a cohort comprised of 21 HPV(+) and 21 HPV(−) tumors provided by Lechner et al*.* [30].

### Cell lines

We investigated DNA methylation of *CCND1*, *FGFR1-4*, *FGF1-14*, and *FGF16-23* in 40 HPV(−) HNSCC cell lines (A253, BB30-HNC, BB49-HNC, BHY, BICR10, BICR22, BICR31, BICR78, Ca9-22, CAL-27, CAL-33, Detroit562, FADU, HN, HO-1-N-1, HO-1-u-1, HSC-2, HSC-3, HSC-4, JHU-011, JHU-022, KON, KOSC-2, LB771-HNC, OSC-19, OSC-20, PCI-15A, PCI-30, PCI-38, PCI-4B, PCI-6A, PE/CA-PJ15, RPMI-2650, SAS, SAT, SCC-15, SCC-25, SCC-4, SCC-9, SKN-3) using a previously published Gene Expression Omnibus (GEO) data set (GEO accession: GSE68379) and the Genomics of Drug Sensitivity in Cancer (GDSC) webpage (https://www.cancerrxgene.org/). Cell line HPV status and *TP53* mutation status (Additional file 2: Table S2) were obtained from GDSC webpage and previously published literature [30]. *TP53* mutated cell lines were considered HPV(−) [8].

### DNA methylation analysis

A total of 840 CpGs within the *FGFR1-4*, *FGF1-14*, *FGF16-23*, and *CCND1* genes and their enclosing sequences were analyzed in this study. DNA methylation data (*β*-values) were generated using the Infinium HumanMethylation450 BeadChip (Illumina, Inc., Diego, CA, USA) technology and downloaded from the UCSC Xena browser (TCGA cohort, www.xena.ucsc.edu) and GEO webpage (GEO accession: GSE68379, HNSCC cell lines; GSE38271 [30]). We considered *β*-values as approximately equal to % methylation. The Infinium HumanMethylation450 BeadChip beads are listed in Additional file 1: Table S1.

### mRNA expression analysis

mRNA expression data were provided by the TCGA Research Network (http://cancergenome.nih.gov/) and were available for *N* = 521 tumor and *N* = 21 NAT samples. Illumina HiSeq 2000 RNA Sequencing Version 2 analysis (Illumina, Inc., San Diego, CA, USA) was used to generate data. Normalized counts per genes were calculated using the SeqWare framework via the RSEM (RNA-Seq by Expectation–Maximization) algorithm. Cell line mRNA expression data (Human Genome U219 Array, Affymetrix, Santa Clara, CA, USA) were downloaded from the ArrayExpress database [31].

### Mutations and copy number variations

Data on *CCND1* and *FGFR1* amplification status of patients’ tumor samples was obtained from The Cancer Genome Atlas Research Network [8]. *CCND1*, FGFR, and FGF copy number variations (CNV) in cell lines as determined using PICNIC algorithms [32] was included from the Cell Modell Passport webpage (Wellcome Sanger Institute, https://cellmodelpassports.sanger.ac.uk/).

### Drug sensitivity [ln(IC_50_)] to FGFR inhibitors

The response data (ln-transformed half maximal inhibitory concentration [ln(IC_50_)]) of HNSCC cell lines to PD 173074 and AZD4547 were obtained from the GDSC database.

### Statistics

SPSS (version 22.0; SPSS Inc., Chicago, IL, USA) and GraphPad Prism 8 software were used for statistical analysis and curves plotting. Mann–Whitney *U* test and analysis of variance (ANOVA) were applied for group comparison of two or more than two groups, respectively. Spearman’s rank correlation (Spearman’s *ρ*) was used for correlation analysis in bivariate analyses. All tests were two-sided, all box plots are depicted with the center line representing the mean. We performed gene-wise Bonferroni correction by multiplication of each *P* value by the number of CpG sites analyzed per gene. *P* values < 0.05 were considered statistically significant. Uncorrected and corrected (*P*_Corrected_) *P* values are reported.

The terms “hyper-” and “hypomethylation” as used herein refer to as statistically significant higher or lower methylation, respectively, compared to a reference group.

## Results

### *CCND1* is differentially methylated between HNSCC tumor and normal adjacent tissues

First, we analyzed DNA methylation of all 79 CpG sites within the gene *CCND1* in tumors and NATs from the TCGA HNSCC cohort. The CpG sites were located in the promoter region as well as the gene body (Additional file 1: Table S1).

**Fig. 1:**
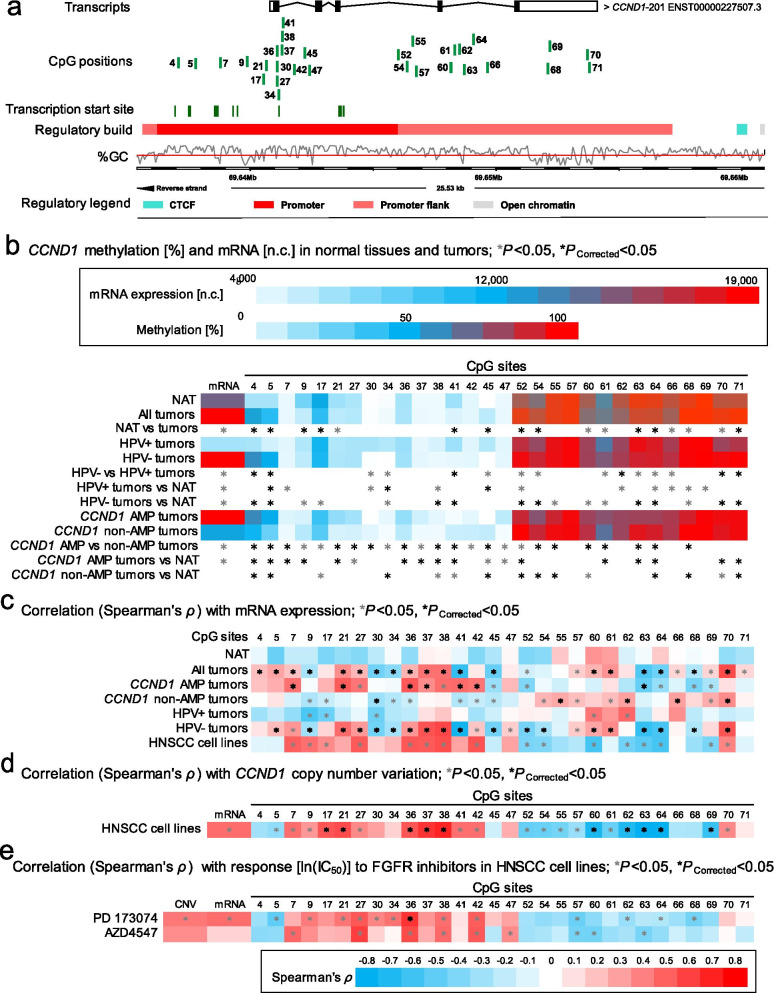
Correlation and association of *CCND1* DNA methylation with mRNA expression, *CCND1* amplification, HPV status, and sensitivity to FGFR-targeted TKIs. **a** Genomic context of 30 selected CpG sites within *CCND1* included into the present study. The illustration, including the predicted regulatory build [63] and transcription start sites according to the Eponine algorithm [64], was exported from www.ensemble.org [65]. **b** Methylation levels of the 30 selected CpG loci within *CCND1* and mRNA expression levels in NATs and tumor tissues, HPV(−) and HPV(+) tumor tissues, and *CCND1* amplified and non-amplified tumors. **c** Spearman’s *ρ* of correlations between *CCND1* DNA methylation and *CCND1* mRNA expression. **d** Spearman’s *ρ* of correlations between *CCND1* DNA methylation and mRNA expression with *CCND1* amplification. **e** Spearman’s *ρ* of correlations of *CCND1* DNA methylation, mRNA expression, and amplification with ln(IC_50_) of PD 173074 and AZD4547. Statistically significant features are marked with asterisks*. *P* values (corrected and uncorrected) refer to Wilcoxon-Mann–Whitney *U* test for comparisons and to Spearman’s *ρ* for correlations analysis, respectively

Regions of low methylation levels were mainly present in the central promoter region of *CCND1* (CpG sites 1–48). 23/79 CpG sites were found to be significantly hypo-methylated in tumor tissue compared to NAT. High methylation levels were predominantly found in the promoter flank region of *CCDN1*. 24/79 CpG sites were significantly hyper-methylated in tumor tissue compared to NAT (CpG sites 52–79). Exemplarily, results from 30 CpG sites are illustrated in Fig. 1a, b.

### FGFRs are differentially methylated between HNSCC tumor and normal adjacent tissues

We further analyzed all CpG sites within the FGF receptors (42 CpG sites within *FGFR1*, 69 within *FGFR2*, 47 within *FGFR3*, and 38 within *FGFR4*). High methylation levels were predominantly found in the gene body (CpG sites 83–95, 122–160, 202–236, and 242–257), while low methylation levels were present in the promoter region (CpG sites 97–113, 166–179, 194–199, and 238; Additional file 1: Table S1).

We compared methylation levels of tumor tissue to methylation levels of NAT within all FGFRs. For *FGFR1* we found significant hyper-methylation at 18/42 and significant hypo-methylation at 16/42 analyzed CpG sites. 8/42 CpG sites were not differentially methylated (Additional file 1: Table S1). Within *FGFR2*, we found 30/69 CpG sites to be significantly hyper-methylated, whereas 11/69 CpGs displayed significant hypo-methylation. No differential methylation was found at 28/69 CpG sites (Additional file 1: Table S1). Within *FGFR3*, we found 23/47 analyzed CpG sites to be hyper-methylated and 5/47 CpG sites to be hypo-methylated. 19/47 CpG sites did not show differential methylation status (Additional file 1: Table S1). Within *FGFR4*, 13/38 CpG sites were hyper-methylated and 19/38 analyzed CpG sites were hypo-methylated. 6/38 CpG sites did not show differential methylation status when comparing tumor tissue to NAT (Additional file 1: Table S1).

**Fig. 2:**
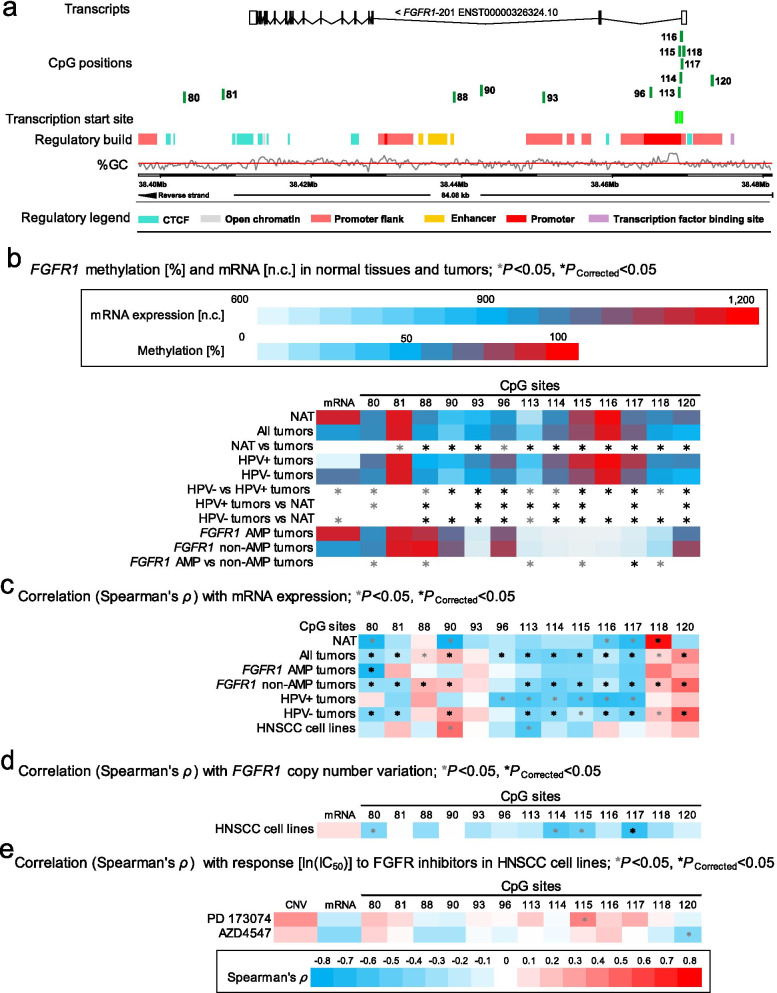
Correlation and association of *FGFR1* DNA methylation with mRNA expression, *FGFR1* amplification, HPV status, and sensitivity to FGFR-targeted TKIs. **a** Genomic context of thirteen selected CpG sites within *FGFR1* included into the present study. The illustration, including the predicted regulatory build [63] and transcription start sites according to the Eponine algorithm [64], was exported from www.ensemble.org [65]. **b** Methylation levels within the thirteen selected CpG loci within *FGFR1* and mRNA expression levels in NAT and tumor tissues, HPV(−) and HPV(+) tumors, and *FGFR1* amplified and non-amplified tumors. **c** Spearman’s *ρ* of correlations between DNA methylation and *FGFR1* mRNA expression. **d** Spearman’s *ρ* of correlations between *FGFR1* DNA methylation and mRNA expression with *FGFR1* amplification. **e** Spearman’s *ρ* of correlations of *FGFR1* DNA methylation, mRNA expression, and amplification with ln(IC_50_) of PD 173074 and AZD4547. Statistically significant features are marked with asterisks *. *P* values (corrected and uncorrected) refer to Wilcoxon-Mann–Whitney *U* test for comparisons and to Spearman’s *ρ* for correlations analysis, respectively

**Fig. 3:**
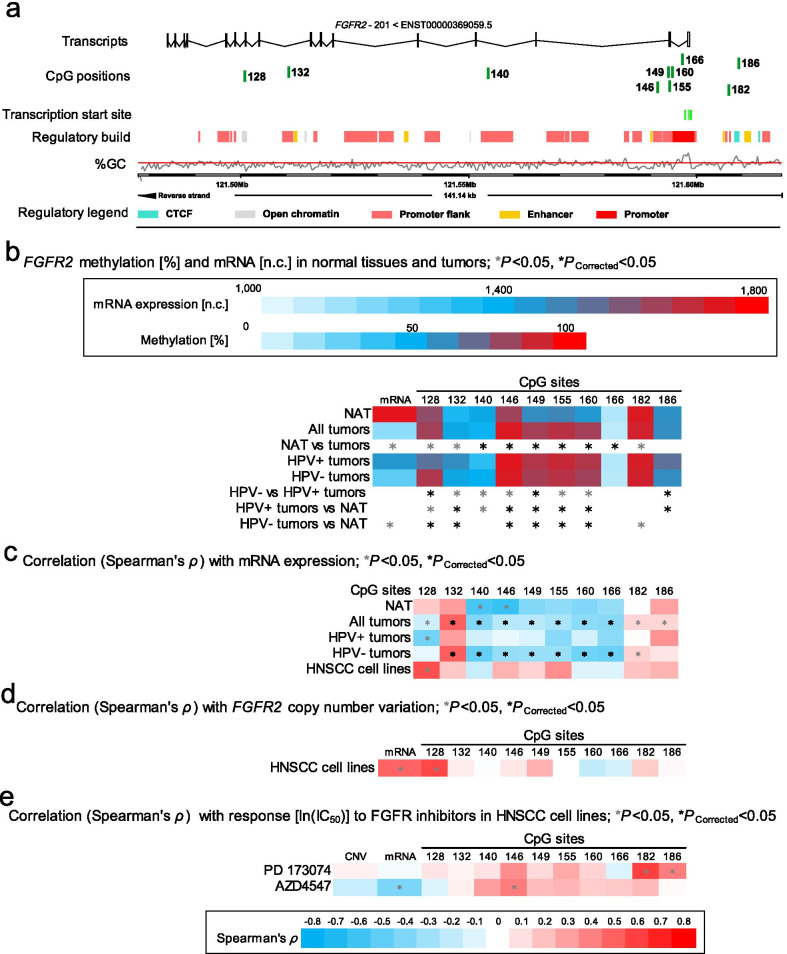
Correlation and association of *FGFR2* DNA methylation with mRNA expression, HPV status, and sensitivity to FGFR-targeted TKIs. **a** Genomic context of ten selected CpG sites within *FGFR2* included into the present study. The illustration, including the predicted regulatory build [63] and transcription start sites according to the Eponine algorithm [64], was exported from www.ensemble.org [65]. **b** Methylation levels within the ten selected CpG loci within *FGFR2* and mRNA expression levels in NAT and tumor tissues and HPV(−) and HPV(+) tumors. **c** Spearman’s *ρ* of correlations between DNA methylation and *FGFR2* mRNA expression. **d** Spearman’s *ρ* of correlations between *FGFR2* DNA methylation and mRNA expression with *FGFR2* amplification. **e** Spearman’s *ρ* of correlations of *FGFR2* DNA methylation, CNV, and mRNA expression with ln(IC_50_) of PD 173074 and AZD4547. Statistically significant features are marked with asterisks *. *P* values (corrected and uncorrected) refer to Wilcoxon-Mann–Whitney *U* test for comparisons and to Spearman’s *ρ* for correlations analysis, respectively

**Fig. 4:**
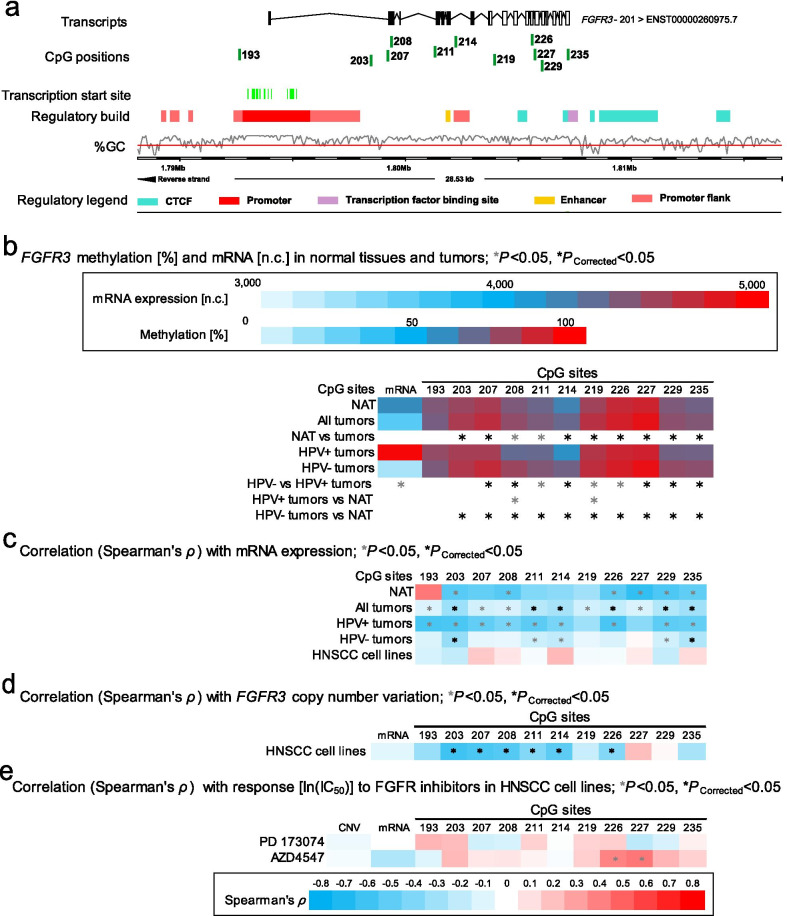
Correlation and association of *FGFR3* DNA methylation with mRNA expression, HPV status, and sensitivity to FGFR-targeted TKIs. **a** Genomic context of eleven selected CpG sites within *FGFR3* included into the present study. The illustration, including the predicted regulatory build [63] and transcription start sites according to the Eponine algorithm [64], was exported from www.ensemble.org [65]. **b** Methylation levels within the eleven selected CpG loci within *FGFR3* and mRNA expression levels in NAT and tumor tissues and HPV(−) and HPV(+) tumors. **c** Spearman’s *ρ* of correlations between DNA methylation and *FGFR3* mRNA expression. **d** Spearman’s *ρ* of correlations between *FGFR3* DNA methylation and mRNA expression with *FGFR3 * amplification. **e** Spearman’s *ρ* of correlations of *FGFR3* DNA methylation, CNV, and mRNA expression with ln(IC_50_) of PD 173074 and AZD4547. Statistically significant features are marked with asterisks *. *P* values (corrected and uncorrected) refer to Wilcoxon-Mann–Whitney *U* test for comparisons and to Spearman’s *ρ* for correlations analysis, respectively

**Fig. 5:**
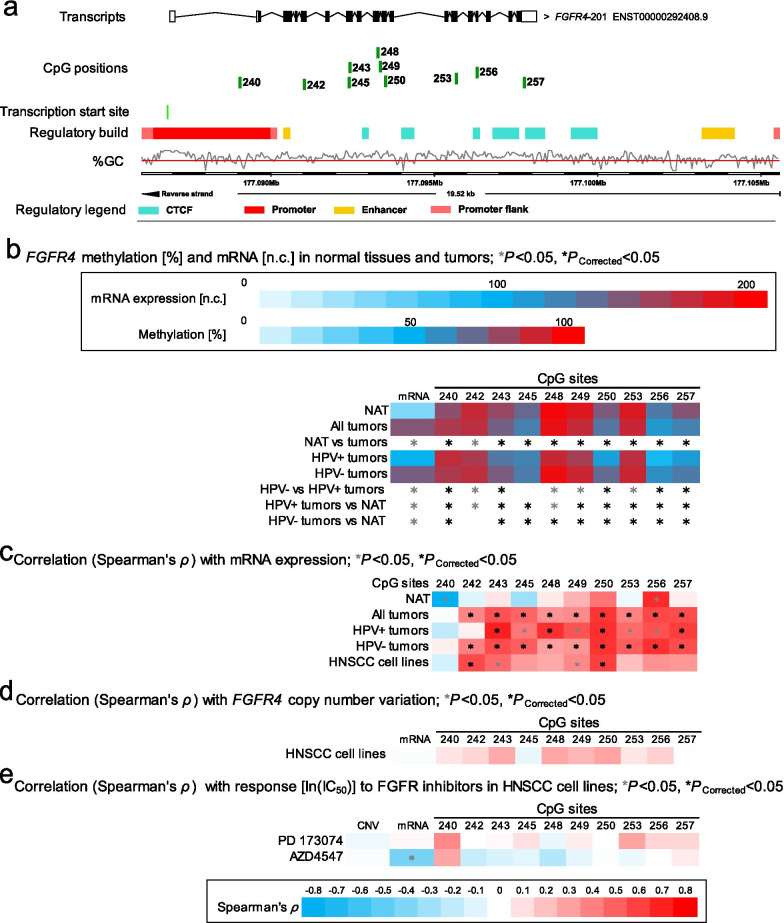
Correlation and association of *FGFR4* DNA methylation with mRNA expression, HPV status, and sensitivity to FGFR-targeted TKIs. **a** Genomic context of ten selected CpG sites within *FGFR4* included into the present study. The illustration, including the predicted regulatory build [63] and transcription start sites according to the Eponine algorithm [64], was exported from www.ensemble.org [65]. **b** Methylation levels within the ten selected CpG loci within *FGFR4* and mRNA expression levels in NAT and tumor tissues and HPV(−) and HPV(+) tumors. **c** Spearman’s *ρ* of correlations between DNA methylation and *FGFR4* mRNA expression. **d** Spearman’s *ρ* of correlations between *FGFR4 *DNA methylation and mRNA expression with *FGFR4 *amplification. **e** Spearman’s *ρ* of correlations of *FGFR4* DNA methylation, CNV, and mRNA expression with ln(IC_50_) of PD 173074 and AZD4547. Statistically significant features are marked with asterisks *. *P* values (corrected and uncorrected) refer to Wilcoxon-Mann–Whitney *U* test for comparisons and to Spearman’s *ρ* for correlations analysis, respectively

Results of selected CpG sites within *FGFR1-4* are illustrated in Fig. 2a, b (*FGFR1*), Fig. 3a, b (*FGFR2*), Fig. 4a, b (*FGFR3*), and Fig. 5a, b (*FGFR4*), respectively.

### FGFs are differentially methylated between HNSCC tumor and normal adjacent tissues

We performed DNA methylation analysis of 565 CpG sites within the FGF genes (no. of analyzed CpGs / FGF gene: 35 / *FGF1*, 19 / *FGF2*, 36 / *FGF3*, 20 / *FGF4*,19 / *FGF5*, 12 / *FGF6*, 7 / *FGF7*, 21 / *FGF8*, 47 / *FGF9*, 15 / *FGF10*, 16 / *FGF11*, 53 / *FGF12*, 52/ *FGF13*, 58 / *FGF14*, 6 / *FGF16*, 13 / *FGF17*, 41 / *FGF18*, 32 / *FGF19*, 22 / *FGF20*, 8 / *FGF21*, 23 / *FGF22*, and 9 / *FGF23*). Among the analyzed CpG sites, 249 were significantly hyper- and 219 hypo-methylated in tumors compared to NAT. Methylation levels depended on the specific location of CpG sites, in detail, the overall promoter methylation level in tumor tissues was significantly higher compared to the overall methylation level in NAT for most of the analyzed CpG sites (Additional file 1: Table S1). 118 CpG sites are displayed in Additional file 3: Fig. S1 (*FGF1*), Additional file 4: Fig. S2 (*FGF2*), Additional file 5: Fig. S3 (*FGF3*, *FGF4*, and *FGF9*), Additional file 6: Fig. S4 (*FGF5*), Additional file 7: Fig. S5 (*FGF6* and *FGF23*), Additional file 8: Fig. S6 (*FGF7*), Additional file 9: Fig. S7 (*FGF8*), Additional file 10: Fig. S8 (*FGF9*), Additional file 11: Fig. S9 (*FGF10*), Additional file 12: Fig. S10 (*FGF11*), Additional file 13: Fig. S11 (*FGF12*), Additional file 14: Fig. S12 (*FGF13*), Additional file 15: Fig. S13 (*FGF14*), Additional file 16: Fig. S14 (*FGF16*), Additional file 17: Fig. S15 (*FGF17*), Additional file 18: Fig. S16 (*FGF18*), Additional file 19: Fig. S17 (*FGF20*), Additional file 20: Fig. S18 (*FGF21*), and Additional file 21: Fig. S19 (*FGF22*), respectively.

### *FGFR*, *FGF*, and *CCND1* methylation is associated with HPV status

We further analyzed DNA methylation levels in tumor tissues with regard to HPV status. For the analyzed CpG sites within *CCND1*, 23/79 were significantly hypo-methylated in HPV(+) tumors compared to HPV(−) tumors. Most of these CpG sites were located in the promoter flank region. 17/79 were hyper-methylated in HPV(+) tumors compared to HPV(−) tumors. The majority of these CpG sites were found to be in the central promoter region (Additional file 1: Table S1, Fig. 1b).

Regarding the CpG sites within *FGFR1*, hyper-methylation was mainly found in HPV(+) tumors: 13/42 were significantly hyper-methylated in HPV(+) tumors compared to HPV(−) tumors in promoter and gene body region; 1/42 (located in an enhancer region) was hypo-methylated in HPV(+) tumors compared to HPV(−) tumors (Additional file 1: Table S1, Fig. 2b).

For *FGFR2*, again, hyper-methylation was mainly found in HPV(+) tumors: 22/69 in the promoter and gene body region were significantly hyper- methylated in HPV(+) tumors compared to HPV(−) tumors; 8/69 in the promoter flank and gene body region were hypo-methylated in HPV(+) tumors compared to HPV(−) tumors (Additional file 1: Table S1, Fig. 3b).

In contrast, *FGFR3* and *FGFR4* hyper-methylation was mainly found in HPV(−) tumors: 30/47 were significantly hyper- and 2/47 hypo-methylated within the *FGFR3* region in HPV(−) tumors compared to HPV(+) tumors; 19/38 were significantly hyper- and 2/38 hypo-methylated in HPV(−) compared to HPV(+) tumors in the *FGFR4* gene region*.* The majority of statistically significant sites are located in the gene body region (Additional file 1: Table S1, Figs. 4b, 5b).

Among the analyzed CpG sites within FGF genes, 195/565 were significantly hyper- and 54/565 significantly hypo-methylated in HPV(−) compared to HPV(+) tumors. High methylation levels were predominantly found in HPV(+) tumors (Additional file 1: Table S1 and Additional file 3: Fig. S1, Additional file 4: Fig. S2, Additional file 5: Fig. S3, Additional file 6: Fig. S4, Additional file 7: Fig. S5, Additional file 8: Fig. S6, Additional file 9: Fig. S7, Additional file 10: Fig. S8 (*FGF9*), Additional file 11: Fig. S9 (*FGF10*), Additional file 12: Fig. S10 (*FGF11*), Additional file 13: Fig. S11 (*FGF12*), Additional file 14: Fig. S12 (*FGF13*), Additional file 15: Fig. S13 (*FGF14*), Additional file 16: Fig. S14 (*FGF16*), Additional file 17: Fig. S15 (*FGF17*), Additional file 18: Fig. S16 (*FGF18*), Additional file 19: Fig. S17 (*FGF20*), Additional file 20: Fig. S18, Additional file 21: Fig. S19).

At last, we validated the methylation differences between HPV(−) and HPV(+) tumors in an independent patient cohort comprised of 42 tumor samples provided by Lechner et al. [30]. Despite the small sample size, we confirmed methylation differences for 112 (29%) out of the 386 CpG sites that showed significant differences in the TCGA cohort (Additional file 1: Table S1).

### *CCND1*, FGFR, and FGF methylation levels correlate with mRNA expression

Regarding *CCND1*, we first analyzed the potential correlation between *CCND1* DNA methylation and mRNA expression in tumor tissue. Significant correlations of DNA methylation levels with mRNA expression were found for most of the analyzed CpG sites (63/79), of which 56% (35/63) showed significant negative correlations. When correlating *CCND1* methylation levels and mRNA expression in NAT, statistically significant correlations was found in only a few sites (7/79) (Fig. 1c).

We analyzed Spearman's correlations between *CCDN1* DNA methylation and *CCND1* mRNA expression in the subgroup of *CCND1* amplified tumors. Methylation of 32/79 CpG sites was significantly correlated to mRNA expression levels. Also, we analyzed *CCND1* DNA methylation and mRNA expression in *CCND1* non-amplified tumors. Here, 42/79 were significantly correlated. Of note, positive correlations were predominantly found in the promoter region and negative correlations within the gene body in *CCND1* amplified tumors, while *CCND1* non-amplified tumors displayed the opposite pattern (Additional file 1: Table S1, Fig. 1c).

We further analyzed the correlations between *FGFR1-4* methylation and mRNA expression. Significant negative correlations are predominantly found in promoter region of *FGFR1* (21/42), while positive correlations are found in the gene body (13/42) (Additional file 1: Table S1, Fig. 2c).

Most analyzed CpG sites within the *FGFR2* (32/69) gene body and promoter showed negative correlations, a few CpG sites (15/69) in gene body and intergenic region exhibited a positive correlation to *FGFR2* mRNA expression (Additional file 1: Table S1, Fig. 3c).

Also, *FGFR3* methylation (32/47) exhibited a negative correlation with *FGFR3* mRNA expression. Most of these CpG sites were located in the gene body region (Additional file 1: Table S1, Fig. 4c).

Furthermore, significant positive correlations between DNA methylation and mRNA expression were found in the gene body of *FGFR4* (21/38), only one CpG site (1/38) located in the promoter region showed a negative correlation with mRNA expression (Additional file 1: Table S1, Fig. 5c).

There was no statistical significance regarding the correlation of DNA methylation of *FGFR1*, *FGFR2*, and *FGFR4* and mRNA expression in NATs. *FGFR3* gene body methylation (21/47) exhibited a negative correlation with *FGFR3* mRNA expression in NATs (Additional file 1: Table S1, Figs. 2c, 3c, 4c, 5c).

In addition, regarding *FGFR1* amplified tumors, only 1/42 CpG sites showed a statistically significant correlation between *FGFR1* DNA methylation and *FGFR1* mRNA expression. *FGFR1* non-amplified tumors, however, exhibited significant negative correlations, which were predominantly found in the promoter region of *FGFR1* (18/42), while positive correlations were found in the gene body (12/42) (Additional file 1: Table S1, Fig. 2c).

Most of the CpG sites within *FGF1* (24/35), *FGF2 (15/19)*, *FGF3* (27/36), *FGF5* (11/19), *FGF7* (6/7), *FGF10* (12/15), *FGF11* (12/16), *FGF12* (45/52), *FGF13* (47/52), *FGF14* (48/58), *FGF18 (23/41)*, *FGF19* (20/32), *FGF20* (17/22), and *FGF21* (8/8) exhibited a statistically significant correlation to mRNA expression of the corresponding gene in tumor tissue with promoter methylation proving to be inversely correlated to mRNA expression. Again, regarding the correlations of DNA methylation with mRNA expression in NATs, only a few CpG sites within *FGF4* (5/20), *FGF6* (1/12), *FGF8* (6/21), *FGF9* (16/47), *FGF16* (1/6), *FGF17* (4/13), and *FGF22* (4/23) displayed significant correlations between mRNA expression and DNA methylation (Additional file 1: Table S1, Additional file 3: Fig. S1, Additional file 4: Fig. S2, Additional file 5: Fig. S3, Additional file 6: Fig. S4, Additional file 7: Fig. S5, Additional file 8: Fig. S6, Additional file 9: Fig. S7, Additional file 10: Fig. S8 (*FGF9*), Additional file 11: Fig. S9 (*FGF10*), Additional file 12: Fig. S10 (*FGF11*), Additional file 13: Fig. S11 (*FGF12*), Additional file 14: Fig. S12 (*FGF13*), Additional file 15: Fig. S13 (*FGF14*), Additional file 16: Fig. S14 (*FGF16*), Additional file 17: Fig. S15 (*FGF17*), Additional file 18: Fig. S16 (*FGF18*), Additional file 19: Fig. S17 (*FGF20*), Additional file 20: Fig. S18, Additional file 21: Fig. S19).

### Correlations of *FGFR1* and *CCND1* gene amplification with methylation and mRNA expression in tumors

*CCND1* gene amplification was significantly associated with increased mRNA expression levels in tumors (*P* < 0.001, Fig. 1b) and cell lines (Fig. 1d). We did not detect increased *FGFR1* mRNA expression levels in *FGFR1* amplified compared to non-amplified tumors (Fig. 2b,* P* = 0.57) or cell lines (Fig. 2d). In HPV(−) cell lines, we further detected a significant positive correlation between *FGFR2* mRNA expression levels and copy number variations (Fig. 3d).

The majority of 79 analyzed CpG sites within *CCND1* showed significantly differential methylation levels between *CCND1* amplified and non-amplified tumors: 21/79 were significantly hyper- and 36/79 hypo-methylated in *CCND1* amplified compared to non-amplified tumors (Additional file 1: Table S1, Fig. 1b). Hyper-methylation was mainly seen within the gene body region, whereas hypo-methylation was found within the central promoter region.

Of note, in HPV(−) cell lines we found significant positive correlations between *CCND1* amplification and methylation in the central promoter region and significant negative correlations within the gene body (Fig. 1d).

Regarding the 42 analyzed CpG sites within *FGFR1,* we detected significant differential methylation between *FGFR1* amplified and non-amplified tumors only in a minority of all CpGs under investigation. 2/42 CpGs were significantly hyper- and 11/42 hypo-methylated in *FGFR1* amplified compared to non-amplified tumors (Additional file 1: Table S1, Fig. 2b).

### Correlations of *CCND1*, FGFR, and FGF DNA methylation, mRNA expression, and amplification in HNSCC cell lines with response to FGFR inhibitors

We first correlated mRNA expression levels with ln(IC_50_) of PD 173074 and AZD4547 in 40 HNSCC cell lines. Among the genes under investigation only *FGF1* expression showed significant negative correlations with IC_50_ of both inhibitors (PD 173074: *ρ* = − 0.38; *P* = 0.021; AZD4547: *ρ* = − 0.38; *P* = 0.022; Additional file 1: Table S1, Additional file 3: Figure S1d).

Next, we analyzed correlations between copy number alterations and ln(IC_50_) of PD 173074 and AZD4547. Copy number alterations did not correlate with ln(IC50) of both inhibitors concomitantly.

**Fig. 6:**
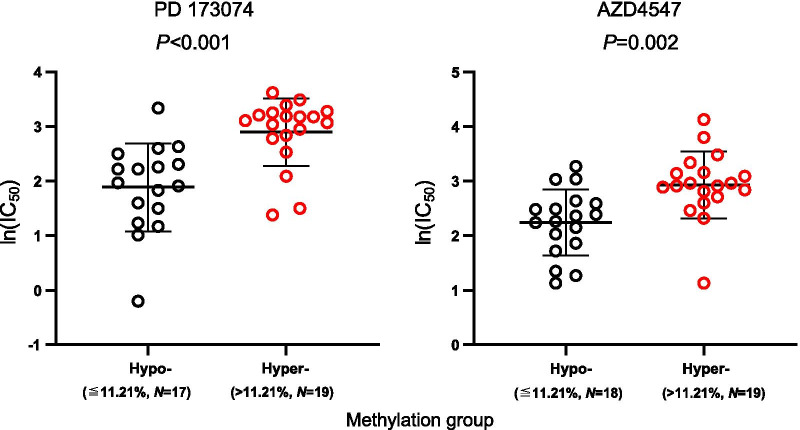
Sensitivity to FGFR inhibitors in *CCND1* (CpG 36) hyper- and hypomethylated HNSCC cell lines. Y-axis represents drug sensitivity [ln(IC_50_)] of HNSCC cell lines treated with FGFR inhibitors PD 173074 and AZD4547. The cell lines were classified as hypo- and hyper-methylated based on the median methylation levels. *P* values refer to Mann–Whitney *U* test

Finally, we evaluated the utility of DNA methylation as a predictive biomarker for response to FGFR inhibitors PD 173074 and AZD4547. A summary of all results can be found in Additional file 1: Table S1. Regarding *CCND1*, methylation of five CpGs (CpGs 27, 36, 38, and 42), located in the upstream central promoter region, was significantly correlated with higher ln(IC_50_) and lower response, respectively, for both inhibitors (Fig. 1e). CpG site 36 within a transcription start site (Fig. 1a) showed the strongest effect (PD 173074: *ρ* = 0.56; *P* < 0.001; AZD4547: *ρ* = 0.48; *P* < 0.001). Methylation of CpG site 57, located in the downstream promoter flank, was negatively correlated with ln(IC_50_) of both TKIs (PD 173074: *ρ* = − 0.41, *P* = 0.013; AZD4547: *ρ* = − 0.37, *P* = 0.023). For better visualization, we dichotomized methylation levels of CpG site 36 using the median methylation level of all cell lines as cut-off. Figure 6 shows that hyper-methylated cell lines responded poorer to both TKIs as indicated by higher ln(IC_50_) levels.

The analyses of all other CpG sites resulted in only two sites that showed significant correlations to ln(IC_50_) levels of both inhibitors concomitantly: CpG site 170, located within the promoter of *FGFR2* (Additional file 1: Table S1)*,* and CpG site 388 within the promoter of *FGF5* (Additional file 6: Figure S4d).

## Discussion

As a stably inherited covalent DNA modification, DNA methylation can be robustly quantified even in fixed and degraded tissues and therefore represents a powerful analytic tool for diagnostic purposes. Moreover, methylation strongly associates with transcriptional gene activity and molecular tumor subtypes, including HPV etiology [26, 33]. However, the value of DNA methylation for prediction of response to TKI is most likely underestimated and so far only poorly investigated. Current knowledge on DNA methylation reveals that DNA methylation patterns are much more nuanced than originally expected [26] and highly depend on the sequence context of the particular CpG site under investigation. While promoter methylation is frequently associated with gene silencing, gene body hyper-methylation is a hallmark of transcriptionally active genes [34, 35]. Consequently, DNA methylation analyses are required to be performed with single CpG site resolution, considering the genomic context of the respective CpG site. This complexity is further increased since other studies describe gene hyper-methylation as an accompanying effect of gene amplification [36]. Gene-expression on protein level is known to be further influenced by additional regulatory mechanisms on mRNA and also protein levels. As a preceding regulatory step, the influence of DNA methylation on the expression of the corresponding protein is poorly understood. We have set our focus on DNA methylation, as it promises to hold different and potentially more significant biological information regarding the activity of a certain pathway and also the possible inhibitability by targeted therapies. In the era of precision medicine, such predictive biomarkers are of immense value since the availability of an increasing number of targeted therapies, which show efficacy only in a small subgroup of patients, necessitates the implementation of companion predictive biomarkers that allow for the precise and accurate stratification of patients eligible for therapy.

Among targeted therapies, the inhibition of FGFRs with the small molecule inhibitor erdafitinib has recently shown high efficacy in heavily pretreated urothelial carcinomas harboring a mutation within FGFRs. This has ultimately led to an FDA breakthrough therapy designation and eventually accelerated approval for the treatment of FGFR-mutated metastatic bladder cancers [11]. Alterations of the FGFR-signaling pathway is a hallmark of HNSCCs and early reports from phase I studies and case-reports suggest efficacy of FGFR-targeted TKIs in HNSCC as well [10, 25, 37]. While mutations, as found in bladder cancers, are rather rare in HNSCC, amplifications of FGFs and FGFRs are among the most common genomic alterations in HNSCC [8]. We and others have previously shown, however, that FGFR amplification does not represent an accurate predictive biomarker for anti-FGFR TKI treatment in HNSCC [38]. This prompted us to investigate DNA methylation of the FGFRs and their ligands with regard to a potential application as a predictive biomarker. Following this path, we describe DNA methylation at single base pair resolution comprising the CpG sites that are covered by the Illumina Infinium HumanMethylation450 BeadChip, taking the complexity of DNA methylation patterns into account.

We investigated methylation of genes involved in FGFR signaling, namely *FGFR1*-*4*, the ligands *FGF1*-*22*, and also *CCND1*, which is co-localized with *FGF3*/*4*/*19* at the 11q13 locus and has also been described to be overexpressed in HNSCC [23]. We analyzed DNA methylation pattern in tumors and normal adjacent tissue and analyzed the association with gene amplification and also with HPV-status. Our results reveal typical features of epigenetically regulated genes. We found significantly differential methylation between tumors and normal adjacent tissues as well as strong associations with HPV infection and gene amplification. Low promoter methylation and high gene body methylation often correlated with increased mRNA expression levels.

Of note, we found positive correlations between *CCND1* mRNA expression and promoter methylation in *CCND1* amplified tumors and negative correlations in non-amplified tumors. Moreover, our study revealed a positive correlation between *CCND1* amplification and *CCND1* methylation in the central promoter region.

Cyclin D1 is a regulator of the G1/S phase transition and is degraded as the cell enters the S phase [39]. Treatment of breast cancer cell lines with the FGFR inhibitor PD 173074 leads to decreased expression of cyclin D1 and a G1 growth arrest [40, 41], hence, providing rationale for cyclin D1 involvement in resistance to FGFR inhibition.

During the cell cycle, DNA methyltransferases (DNMTs) are differentially expressed [42]. Expression levels of the de novo DNMT3b shows it lowest levels during G1 phase and peaks in the late S phase [42]. DNMT3a mediated de novo methylation also requires cell division [43]. Consequently, DNA methylation pattern have been shown to vary during a single cell cycle. Global DNA methylation levels decrease during the G1 and increase in the S phase [44]. Taken together, these findings suggest that aberrant cyclin D1 overexpression caused by *CCND1* gene amplification could lead to hypermethylation, potentially also affecting the *CCND1* gene locus. In line, *CCND1* amplification has been reported to be associated with a CpG island methylator phenotype (CIMP)-high status in breast and colon cancer [45, 46].

HPV infection is an important susceptibility factor for HNSCC and is directly involved in the pathogenesis, especially in oropharyngeal squamous cell carcinoma [47, 48]. Minarovits et al*.* comprehensively reviewed how oncoproteins encoded by human tumor viruses significantly interact with the cellular epigenetic machinery and alter the epigenome of both host cells and the virus itself, in order to overcome host’s immune response and promote their own viral replication [49]. Hence, it is not surprising that virus-associated tumors exhibit a distinct CpG methylation pattern. Brennan et al*.* defined five HNSCCs methylation subtypes: one HPV(+) subtype, two smoking-related subtypes, and two atypical subtypes [33]. Our results also show an association of genomic and epigenomic FGFR signaling pathway alterations with HPV infection status. We found FGFRs, FGFs, and *CCND1* to be differentially methylated between HPV(+) and HPV(−) tumors. *FGFR1-2* and FGFs hyper-methylation was observed in HPV(+) tumors, which is consistent with previous studies and findings [50, 51].

We further investigated correlations between CpG methylation and response to two FGFR inhibitors (PD 173074 and AZ4547) in 40 HPV(−) HNSCC cell lines and provide first evidence of an association of CpG methylation with response to TKI. PD 173074 is a selective small molecule FGFR1/3 TKI disrupting FGFR family related signaling [52, 53]. PD 173074 significantly reduces the proliferation of triple negative breast cancer cell SUM185PE (harboring a *FGFR3-TACC3* fusion) and also seems to be involved in cell cycle arrest within the G1 phase, apoptosis, and decrease of fibroblast growth factor receptor substrate 2 (FRS2) and AKT activation [54]. Studies of selective FGFR inhibitors reported tumor cell lines with high *FGFR1*/*3* expression to be more sensitive to the *FGFR1/3* inhibitor PD 173074 [55]. AZD4547 is a potent inhibitor of FGFR1-3 with weaker activity against FGFR4 and other kinases [56].

In our study, we found DNA methylation of nine CpG sites within *FGF5*, *FGFR2*, and *CCND1* correlating with response to PD 173074 and AZD4547. Interestingly, although PD 173074 and AZD4547 are selective FGFR inhibitors, we found most CpG sites with significant correlation to drug response to be located within *CCND1*. Only one significant CpG site was located within a FGF receptor (*FGFR2*). This also further illustrates the diversity of epigenetic regulation. Thus, in addition to DNA methylation, e.g. microRNAs and posttranslational modifications have to be taken into account, leading to the need of further investigations following the results of this present study. However, since the analyzed cell lines are HPV(−), the transferability of our results to HPV(+) cell lines needs to be tested in further studies.

So far, patients for FGFR inhibitor treatment are selected based on *FGFR1* gene amplification levels, *FGFR3* mutations, or *FGFR2*/*FGFR3* fusions [57–60]. Previous studies displayed that *FGFR1* gene amplification does not necessarily induce higher mRNA levels and also does not give adequate prognosis as to sensitivity to TKIs [38]. Our study suggests methylation as a novel biomarker for patient selection, although further studies are required to define which gene and which CpG site is of highest significance. Epigenetic features such as methylation could play a dominant role in repressing amplified genes, so we took the next step towards biomarker-driven personalized treatment options by describing gene methylation in relation to amplification regarding the predictability of response to TKI treatment in HNSCC.

In this study, we aim to provide a rationale for testing DNA methylation as a predictive biomarker for response to anti-FGFR TKI in HNSCC. The high number of analyzed patients’ samples that allows for the detection of even minor differences and weak correlations that are statistically significant is a strength of our study. However, the biological relevance of such findings remains unclear and needs further investigation. Further validation needs to be performed in a HNSCC patient cohort treated with anti-FGFR TKIs. Unfortunately, an adequately sized cohort allowing for a sufficiently powered analysis is currently not available. We were able to describe a detailed picture of DNA methylation pattern in HNSCC by analyzing multiple CpG sites, thus enabling a pre-selection of specific CpG sites of interest for further validation. We reported uncorrected and Bonferroni-corrected *P* values even though several CpG sites are located in close proximity to each other and therefore contain redundant biological information. Therefore, testing these adjacent CpG sites does not necessarily reflect multiple testing and correction for multiple testing might result in overall too pessimistic results. However, our study provides a preselection of CpG sites for further validation in an FGFR TKI treated validation cohort and therefore will reduce the problem of multiple testing in such a validation study. The low CpG coverage of the employed Illumina HumanMethylation450 BeadChip, however, is a limitation of our study. The BeadChip covers appr. 450,000 CpG sites out of the ~ 36 million CpGs (~ 1.25%) that are present in the whole genome. Even in genomic regions with an enriched CpG coverage, as present per design in promoter regions, the coverage is too low to allow for definite conclusions. Bisulfite sequencing based methods for methylation analysis at single CpG site resolution need to be employed in future studies in order to provide a complete picture of the methylation landscape. The high performance of next generation sequencing methods could further be exploited to combine methylation testing with mutation testing for an accurate response prediction or for an internal normalization for tumor cell content in a clinical sample.

In the current study, we only employed two selective FGFR inhibitors, which failed in clinical studies und thus represents a limitation of our study. AZD4547, for example, revealed low tolerability and low efficacy (NCT04439240, NCT02965378). However, since the mode of action of selective TKI inhibitors is similar [61, 62], our results could have general validity, which will have to be shown in additional studies employing other selective TKIs. In particular, this validation should also be performed in a patient cohort receiving selective anti-FGFR inhibitors that are currently in clinical development, e.g. edrafitinib, rogaratinib, infigratinib, pemigatinib or futibatinib.

## Conclusions

In conclusion, our study shows a sequence-contextually nuanced DNA methylation pattern of *CCND1*, FGFRs, and FGFs that associates with mRNA expression levels, gene amplification status, and sensitivity to selective FGFR inhibition in HNSCC. These findings suggest an epigenetic gene regulation by DNA methylation. Accordingly, our study provides a rationale to include methylation analysis, particularly of *CCND1*, into companion biomarker programs of clinical trials testing FGFR-directed TKIs in HNSCC.

## Supplementary Information

**Additional file 1: Table S1.** DNA methylation analysis of the encoding genes *CCND1*, *FGFR1-4*, *FGF1-14*, and *FGF16-23* at single CpG site resolution (840 CpG sites), employing The Cancer Genome Research Atlas (TCGA) HNSCC cohort (*N* = 530 tumor and *N* = 50 normal adjacent tissue samples) and HNSCC cell lines (N=40). Single CpG DNA methylation level, spearman’s rank correlation to mRNA expression with regard to human papilloma (HPV) and gene amplification status, and correlation of methylation with sensitivity to the selective FGFR inhibitors PD 173074 and AZD4547 in HNSCC cell lines were included.

**Additional file 2: Table S2.** DNA methylation, copy number variation, drug sensitivity, *TP53* mutation status, and HPV-status of analyzed HNSCC cell lines.

**Additional file 3: Fig. S1.** This figure illustrates correlation and association of *FGF1* DNA methylation with mRNA expression, HPV status, copy number variation, and sensitivity to the FGFR-targeted TKIs PD 173074 and AZD4547. Exemplarily, results of seven selected CpG sites within *FGF1* are illustrated.

**Additional file 4: Fig. S2.** This figure illustrates correlation and association of *FGF2* DNA methylation with mRNA expression, HPV status, copy number variation, and sensitivity to the FGFR-targeted TKIs PD 173074 and AZD4547. Exemplarily, results of six selected CpG sites within *FGF2* are illustrated.

**Additional file 5: Fig. S3.** This figure illustrates correlation and association of *FGF19*, *FGF4,* and *FGF3* DNA methylation with mRNA expression, HPV status, copy number variation, and sensitivity to the FGFR-targeted TKIs PD 173074 and AZD4547. Exemplarily, results of 11 selected CpG sites within *FGF19*, *FGF4*; and *FGF3* are illustrated.

**Additional file 6: Fig. S4.** This figure illustrates correlation and association of *FGF5* DNA methylation with mRNA expression, HPV status, copy number variation, and sensitivity to the FGFR-targeted TKIs PD 173074 and AZD4547. Exemplarily, results of three selected CpG sites within *FGF5* are illustrated.

**Additional file 7: Fig. S5.** This figure illustrates correlation and association of *FGF23* and *FGF6* DNA methylation with mRNA expression, HPV status, copy number variation, and sensitivity to the FGFR-targeted TKIs PD 173074 and AZD4547. Exemplarily, results of five selected CpG sites within *FGF23* and *FGF6* are illustrated.

**Additional file 8: Fig. S6.** This figure illustrates correlation and association of *FGF7* DNA methylation with mRNA expression, HPV status, copy number variation, and sensitivity to the FGFR-targeted TKIs PD 173074 and AZD4547. Exemplarily, results of three selected CpG sites within *FGF7* are illustrated.

**Additional file 9: Fig. S7.** This figure illustrates correlation and association of *FGF8* DNA methylation with mRNA expression, HPV status, and copy number variation, sensitivity to the FGFR-targeted TKIs PD 173074 and AZD4547. Exemplarily, results of four selected CpG sites within *FGF8* are illustrated.

**Additional file 10: Fig. S8.** This figure illustrates correlation and association of *FGF9* DNA methylation with mRNA expression, HPV status, copy number variation, and sensitivity to the FGFR-targeted TKIs PD 173074 and AZD4547. Exemplarily, results of eight selected CpG sites within *FGF9* are illustrated.

**Additional file 11: Fig. S9.** This figure illustrates correlation and association of *FGF10* DNA methylation with mRNA expression, HPV status, copy number variation, and sensitivity to the FGFR-targeted TKIs PD 173074 and AZD4547. Exemplarily, results of 4 selected CpG sites within *FGF10* are illustrated.

**Additional file 12: Fig. S10.** This figure illustrates correlation and association of *FGF11* DNA methylation with mRNA expression, HPV status, copy number variation, and sensitivity to the FGFR-targeted TKIs PD 173074 and AZD4547. Exemplarily, results of eight selected CpG sites within *FGF11* are illustrated.

**Additional file 13: Fig. S11.** This figure illustrates correlation and association of *FGF12* DNA methylation with mRNA expression, HPV status, copy number variation, and sensitivity to the FGFR-targeted TKIs PD 173074 and AZD4547. Exemplarily, results of 12 selected CpG sites within *FGF12* are illustrated.

**Additional file 14: Fig. S12.** This figure illustrates correlation and association of *FGF13* DNA methylation with mRNA expression, HPV status, copy number variation, and sensitivity to the FGFR-targeted TKIs PD 173074 and AZD4547. Exemplarily, results of five selected CpG sites within *FGF13* are illustrated.

**Additional file 15: Fig. S13.** This figure illustrates correlation and association of *FGF14* DNA methylation with mRNA expression, HPV status, copy number variation, and sensitivity to the FGFR-targeted TKIs PD 173074 and AZD4547. Exemplarily, results of ten selected CpG sites within *FGF14* are illustrated.

**Additional file 16: Fig. S14.** This figure illustrates correlation and association of *FGF16* DNA methylation with mRNA expression, HPV status, copy number variation, and sensitivity to the FGFR-targeted TKIs PD 173074 and AZD4547. Exemplarily, results of six selected CpG sites within *FGF16* are illustrated.

**Additional file 17: Fig. S15.** This figure illustrates correlation and association of *FGF17* DNA methylation with mRNA expression, HPV status, copy number variation, and sensitivity to the FGFR-targeted TKIs PD 173074 and AZD4547. Exemplarily, results of seven selected CpG sites within *FGF17* are illustrated.

**Additional file 18: Fig. S16.** This figure illustrates correlation and association of *FGF18* DNA methylation with mRNA expression, HPV status, copy number variation, and sensitivity to the FGFR-targeted TKIs PD 173074 and AZD4547. Exemplarily, results of 12 selected CpG sites within *FGF18* are illustrated.

**Additional file 19: Fig. S17.** This figure illustrates correlation and association of *FGF20* DNA methylation with mRNA expression, HPV status, copy number variation, and sensitivity to the FGFR-targeted TKIs PD 173074 and AZD4547. Exemplarily, results of three selected CpG sites within *FGF20* are illustrated.

**Additional file 20: Fig. S18.** This figure illustrates correlation and association of *FGF21* DNA methylation with mRNA expression, HPV status, copy number variation, and sensitivity to the FGFR-targeted TKIs PD 173074 and AZD4547. Exemplarily, results of eight selected CpG sites within *FGF21* are illustrated.

**Additional file 21: Fig. S19.** This figure illustrates correlation and association of *FGF22* DNA methylation with mRNA expression, HPV status, copy number variation, and sensitivity to the FGFR-targeted TKIs PD 173074 and AZD4547. Exemplarily, results of eight selected CpG sites within *FGF22* are illustrated.

## Data Availability

The datasets of DNA methylation, copy number variation, and mRNA expression used in the current study are available from the corresponding author on reasonable request, from TCGA Research Network (http://cancergenome.nih.gov/), from the Cell Modell Passport webpage (Wellcome Sanger Institute, https://cellmodelpassports.sanger.ac.uk/), from the ArrayExpress database, GDSC webpage (https://www.cancerrxgene.org/) or can be downloaded from Gene Expression Omnibus (GEO accession: GSE68739 and GSE38271).

## Abbreviations

ACC: Adenoid cystic carcinoma
AKT: Protein kinase B
ANOVA: Analysis of variance
CCND1: Cyclin D1
CNV: Copy number variation
EBV: Epstein-Barr virus
EC: Extracellular
EGFR: Epidermal growth factor receptor
ERBB2: Human epidermal growth factor receptor 2
FDA: Food and Drug Administration
FGF: Fibroblast growth factor
FGFR: Fibroblast growth factor receptor
FRS2: Fibroblast growth factor receptor substrate 2
GEO: Gene Expression Omnibus
HNSCC: Head and neck squamous cell carcinoma
HPV: Human papillomavirus
HPV(+): HPV-positive
HPV(–): HPV-negative
HR: Hazard ratio
MGMT: O6-methylguanine–DNA methyltransferase
NAT: Normal adjacent tissue
NPC: Nasopharyngeal carcinoma
OS: Overall survival
PFS: Progression-free survival
RNA-Seq: RNA sequencing
RSEM: RNA-Seq by Expectation–Maximization
RTK: Receptor tyrosine kinase
Spearman´s *ρ*: Spearman´s rank correlation
TCGA: The Cancer Genome Research Atlas
TKI: Tyrosine kinase inhibitor
VEGFR: Vascular endothelial growth factor receptor
95% CI: 95% Confidence interval
