# Supplementary material for: Fibroblast growth factor (FGF), FGF receptor (FGFR), and cyclin D1 (CCND1) DNA methylation in head and neck squamous cell carcinomas is associated with transcriptional activity, gene amplification, human papillomavirus (HPV) status, and sensitivity to tyrosine kinase inhibitors
Source: Clin Epigenetics. 2021 Dec 21;13:228. doi: 10.1186/s13148-021-01212-4 (PMC8693503; doi:10.1186/s13148-021-01212-4)
Supplement: Supplementary file 3 — Additional file 3: Fig. S1. This figure illustrates correlation and association of FGF1 DNA methylation with mRNA expression, HPV status, copy number variation, and sensitivity to the FGFR-targeted TKIs PD 173074 and AZD4547. Exemplarily, results of seven selected CpG sites within FGF1 are illustrated. [file 13148_2021_1212_MOESM3_ESM.pdf]

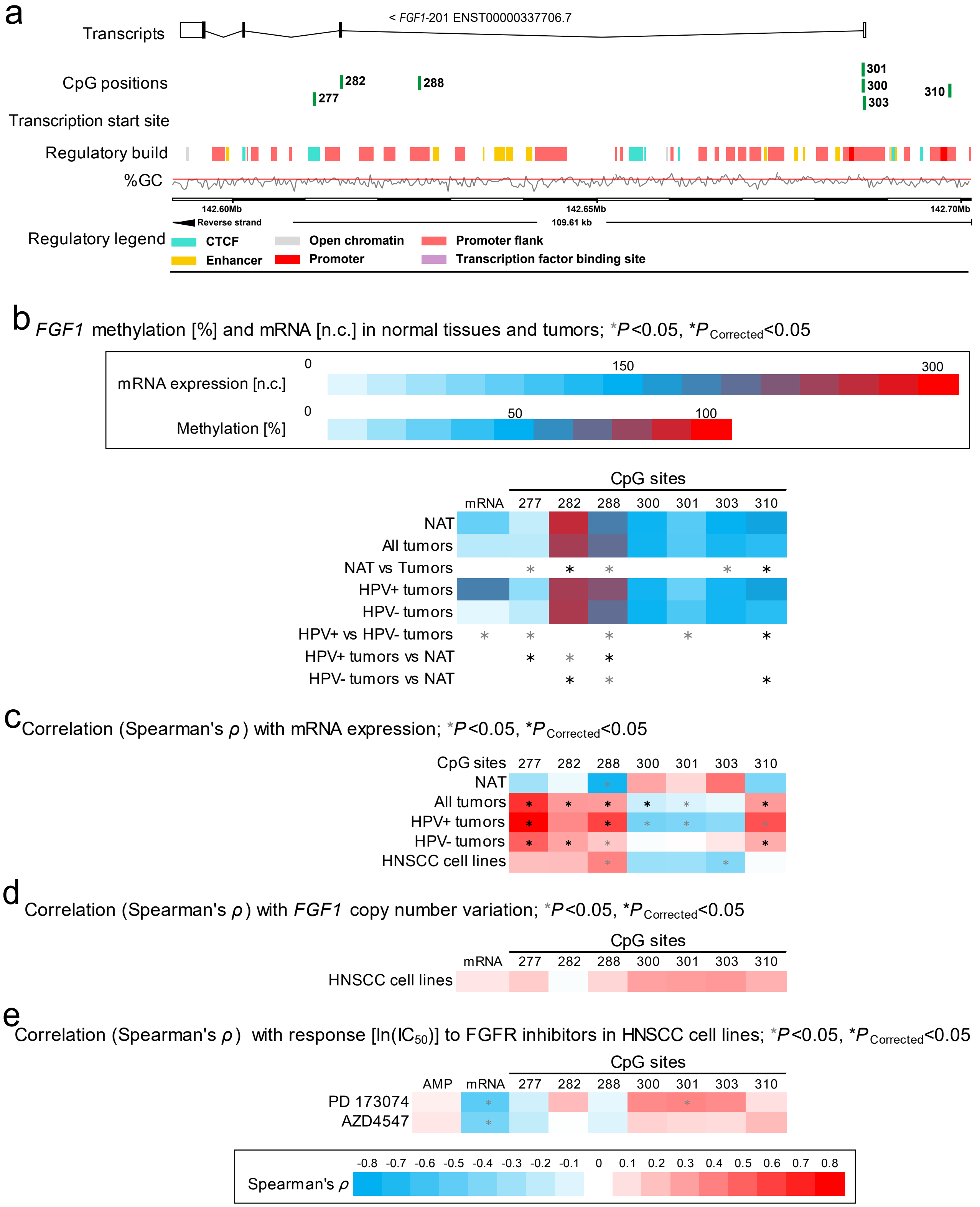

a
Transcripts
< FGF1-201 ENST00000337706.7
CpG positions
282
288
277
301
300
303
310
Transcription start site
Regulatory build
%GC
142.60Mb
142.65Mb
142.70Mb
Reverse strand
109.61 kb
Regulatory legend
CTCF
Enhancer
Open chromatin
Promoter
Promoter flank
Transcription factor binding site
b
FGF1 methylation [%] and mRNA [n.c.] in normal tissues and tumors; *P<0.05, *P Corrected<0.05
mRNA expression [n.c.]
0
150
300
Methylation [%]
0
50
100
CpG sites
mRNA 277 282 288 300 301 303 310
NAT
All tumors
NAT vs Tumors
HPV+ tumors
HPV- tumors
HPV+ vs HPV- tumors
HPV+ tumors vs NAT
HPV- tumors vs NAT
c
Correlation (Spearman's ρ) with mRNA expression; *P<0.05, *P Corrected<0.05
CpG sites 277 282 288 300 301 303 310
NAT
All tumors
HPV+ tumors
HPV- tumors
HNSCC cell lines
d
Correlation (Spearman's ρ) with FGF1 copy number variation; *P<0.05, *P Corrected<0.05
CpG sites
mRNA 277 282 288 300 301 303 310
HNSCC cell lines
e
Correlation (Spearman's ρ) with response [ln(IC50)] to FGFR inhibitors in HNSCC cell lines; *P<0.05, *P Corrected<0.05
CpG sites
AMP mRNA 277 282 288 300 301 303 310
PD 173074
AZD4547
Spearman's ρ
-0.8 -0.7 -0.6 -0.5 -0.4 -0.3 -0.2 -0.1 0 0.1 0.2 0.3 0.4 0.5 0.6 0.7 0.8
