# Supplementary figures and images for: Fibroblast growth factor (FGF), FGF receptor (FGFR), and cyclin D1 (CCND1) DNA methylation in head and neck squamous cell carcinomas is associated with transcriptional activity, gene amplification, human papillomavirus (HPV) status, and sensitivity to tyrosine kinase inhibitors
Source: Clin Epigenetics. 2021 Dec 21;13:228. doi: 10.1186/s13148-021-01212-4 (PMC8693503; doi:10.1186/s13148-021-01212-4)

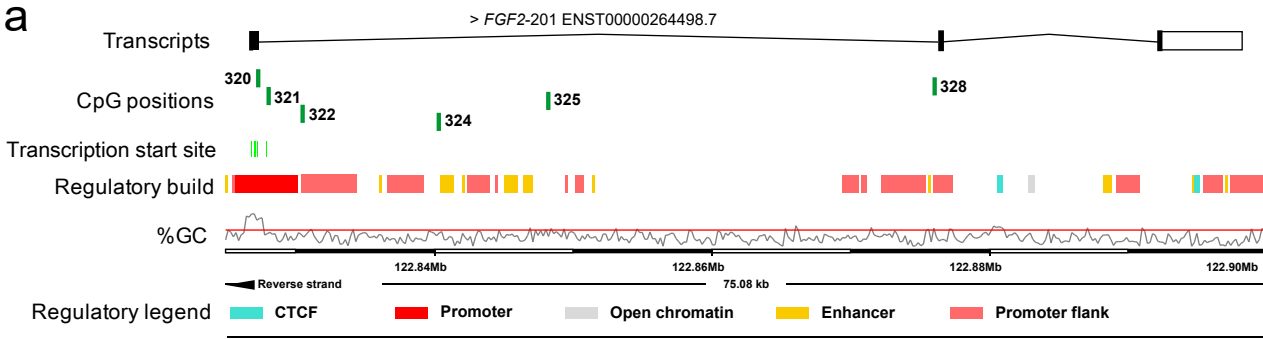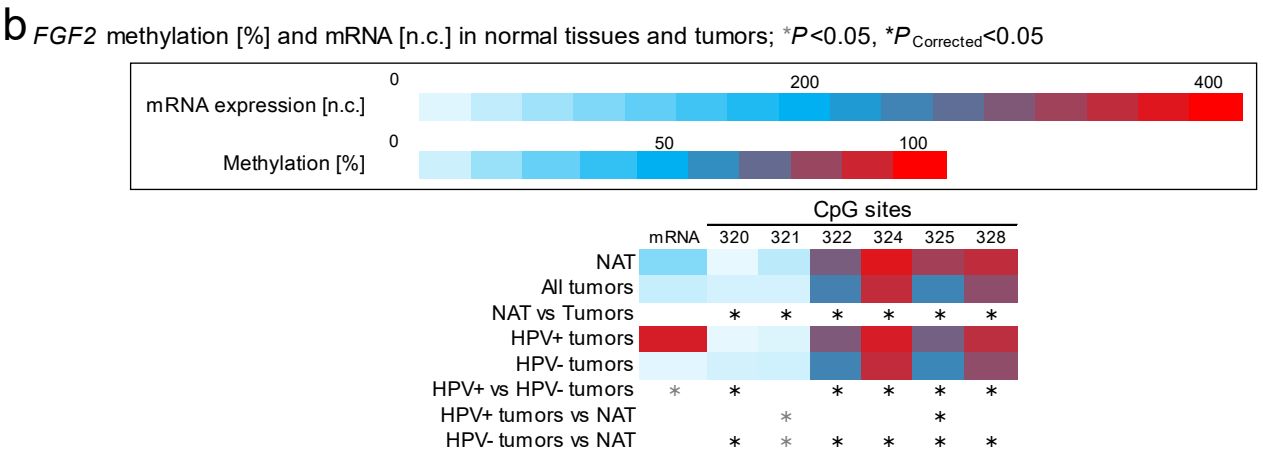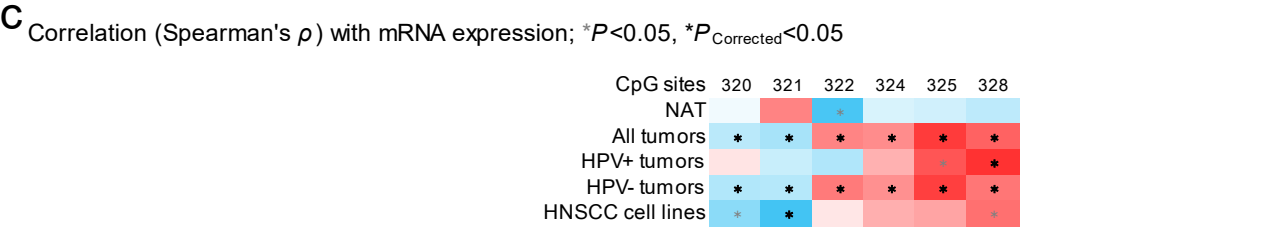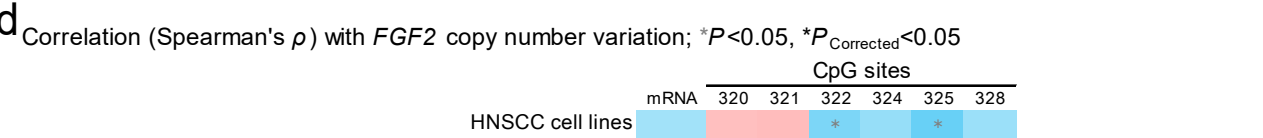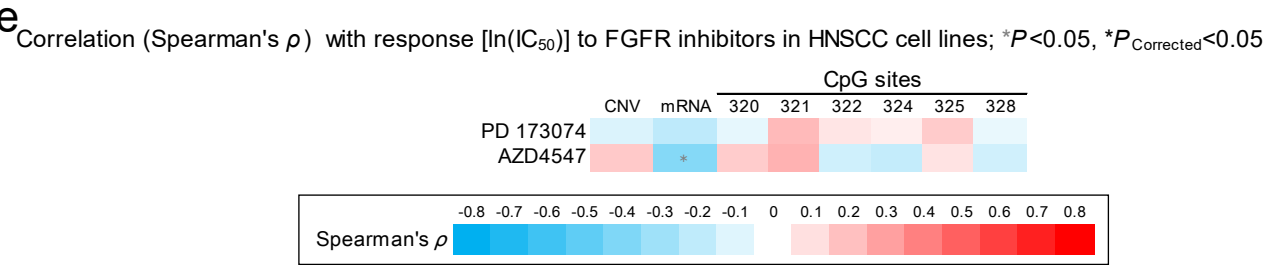

Supplement: Supplementary file 4 — Additional file 4: Fig. S2. This figure illustrates correlation and association of FGF2 DNA methylation with mRNA expression, HPV status, copy number variation, and sensitivity to the FGFR-targeted TKIs PD 173074 and AZD4547. Exemplarily, results of six selected CpG sites within FGF2 are illustrated. [file 13148_2021_1212_MOESM4_ESM.pdf]

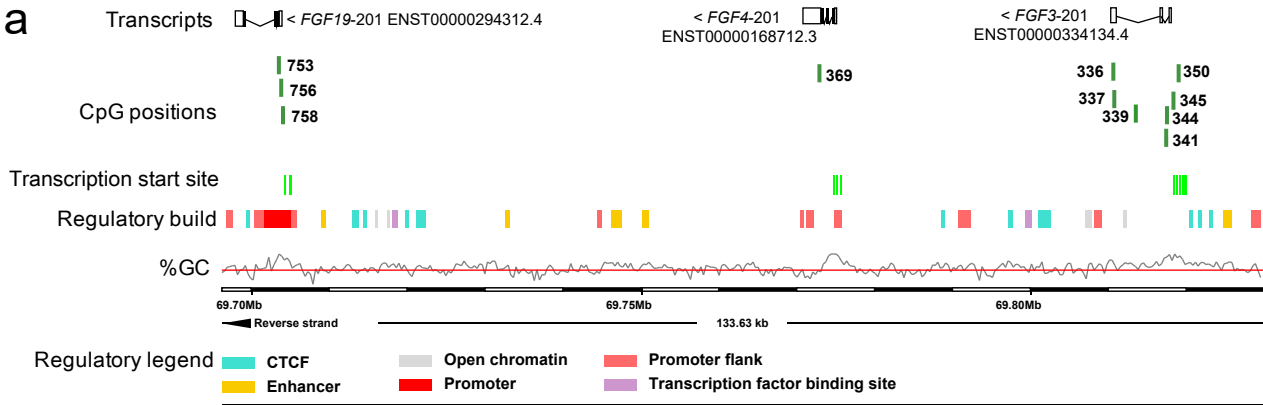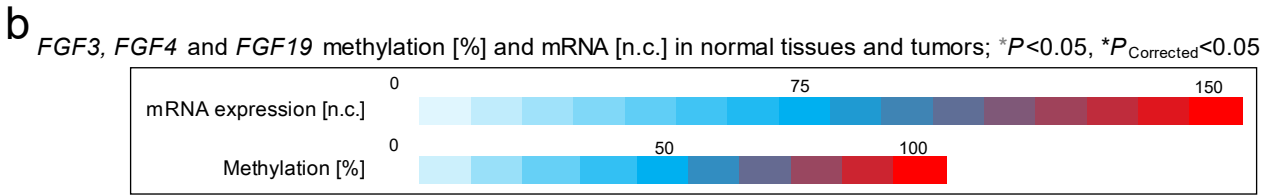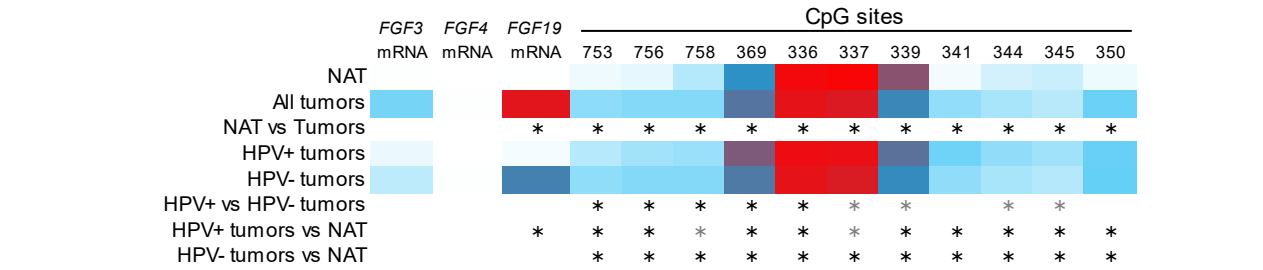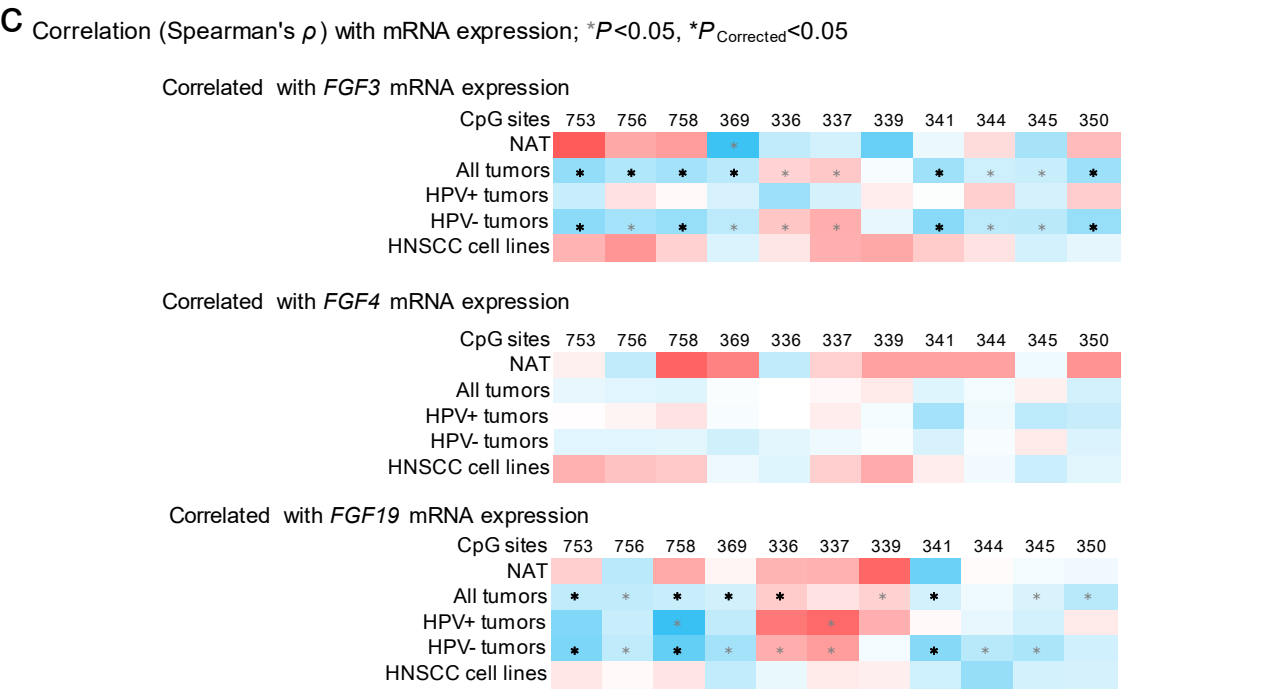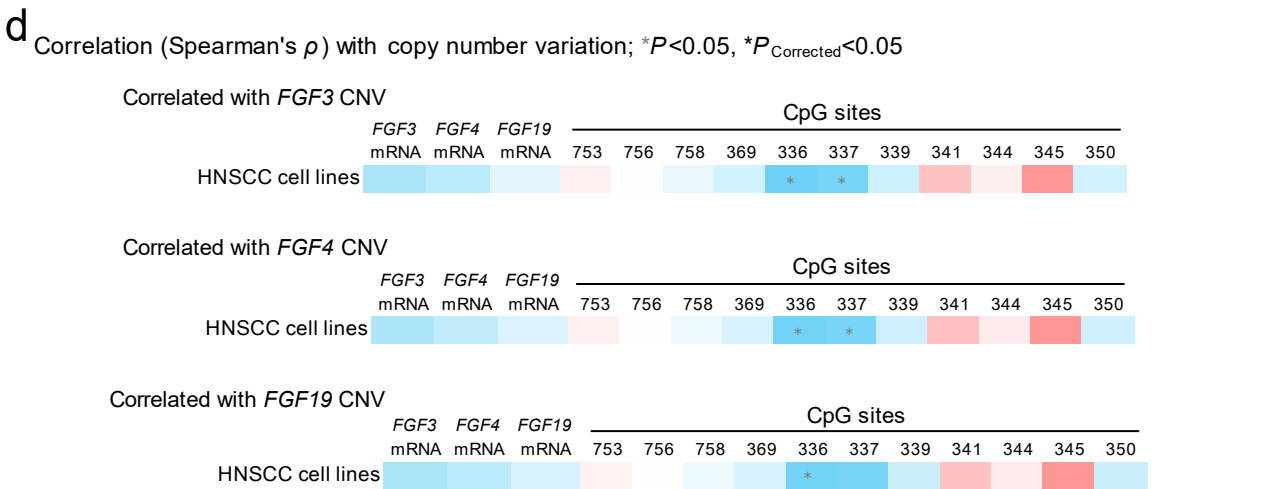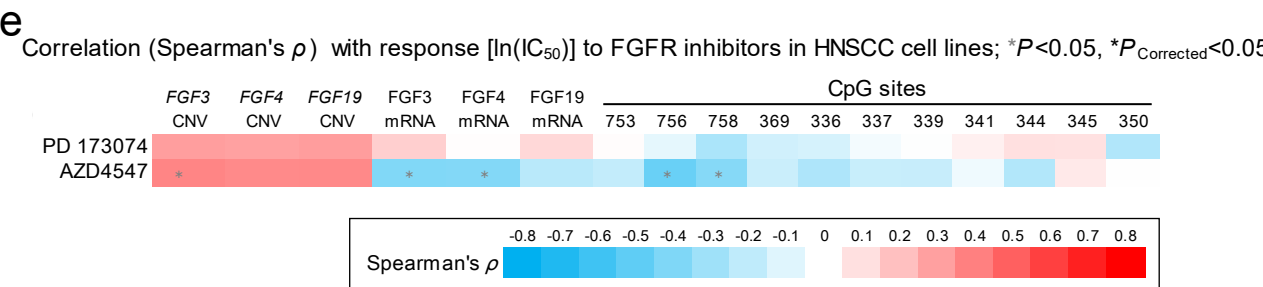

Supplement: Supplementary file 5 — Additional file 5: Fig. S3. This figure illustrates correlation and association of FGF19, FGF4, and FGF3 DNA methylation with mRNA expression, HPV status, copy number variation, and sensitivity to the FGFR-targeted TKIs PD 173074 and AZD4547. Exemplarily, results of 11 selected CpG sites within FGF19, FGF4; and FGF3 are illustrated. [file 13148_2021_1212_MOESM5_ESM.pdf]

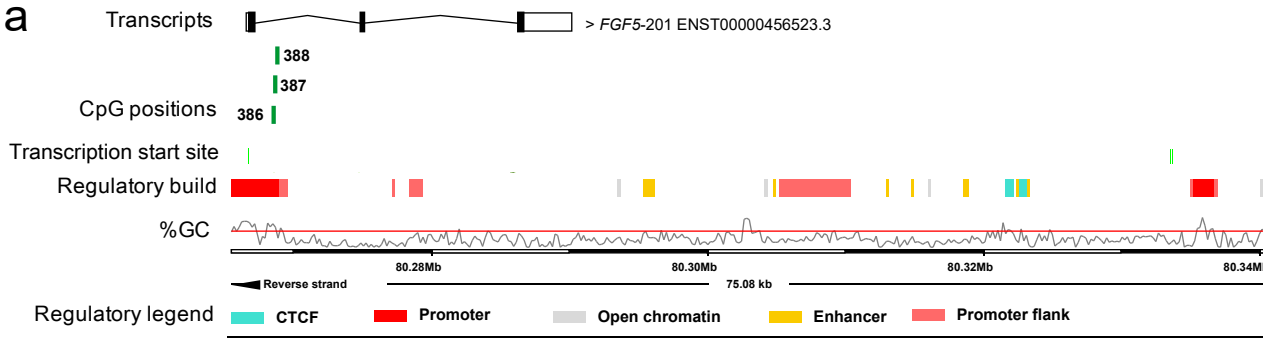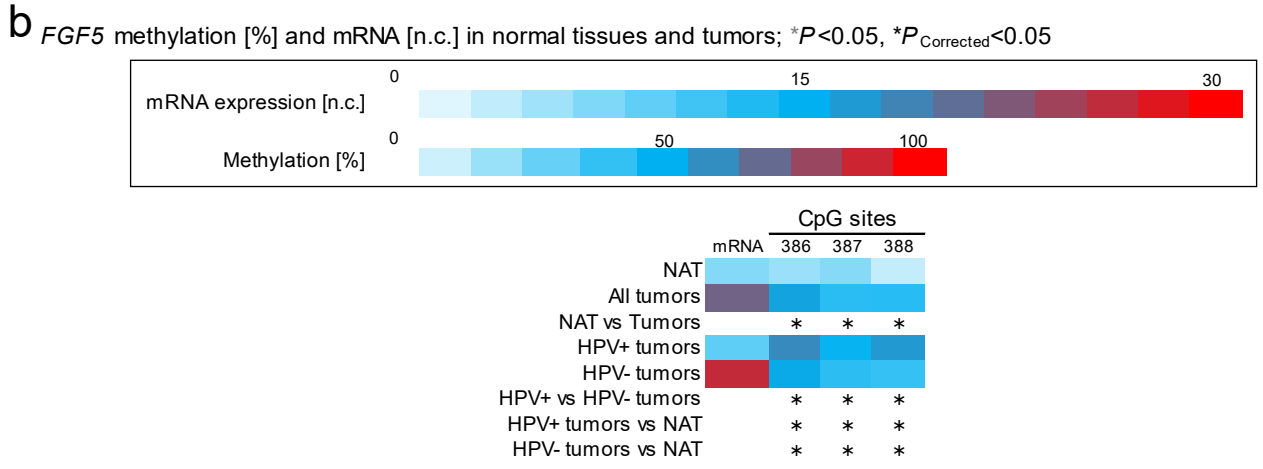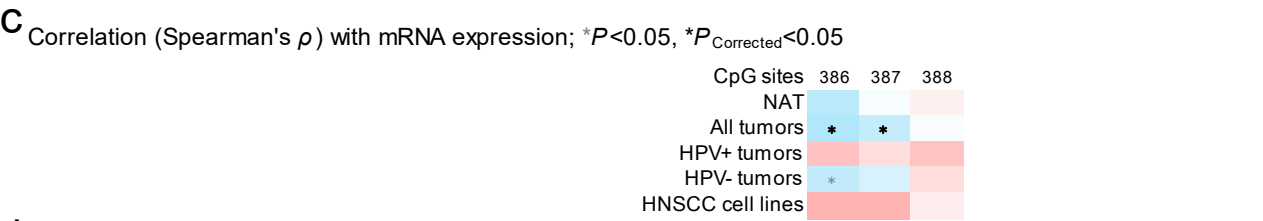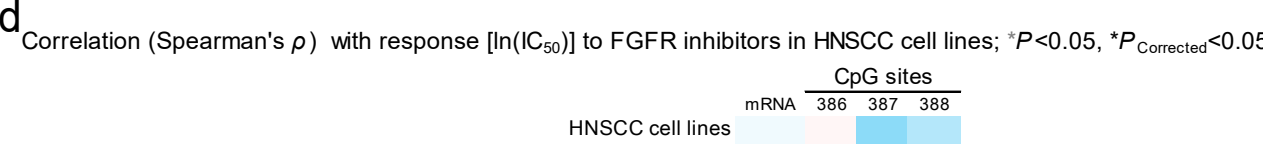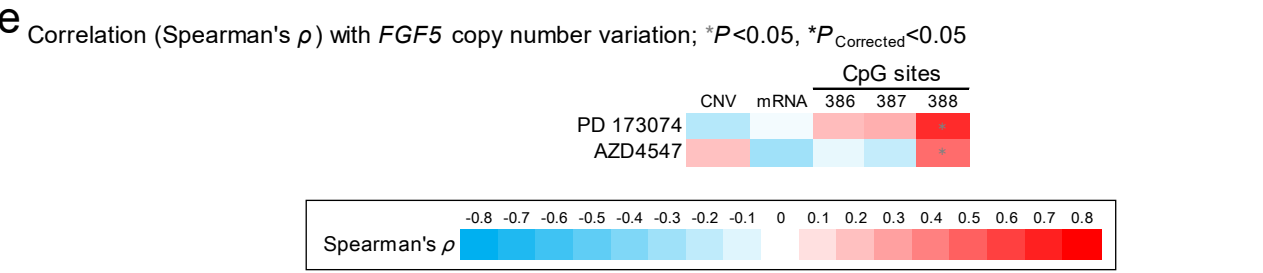

Supplement: Supplementary file 6 — Additional file 6: Fig. S4. This figure illustrates correlation and association of FGF5 DNA methylation with mRNA expression, HPV status, copy number variation, and sensitivity to the FGFR-targeted TKIs PD 173074 and AZD4547. Exemplarily, results of three selected CpG sites within FGF5 are illustrated. [file 13148_2021_1212_MOESM6_ESM.pdf]

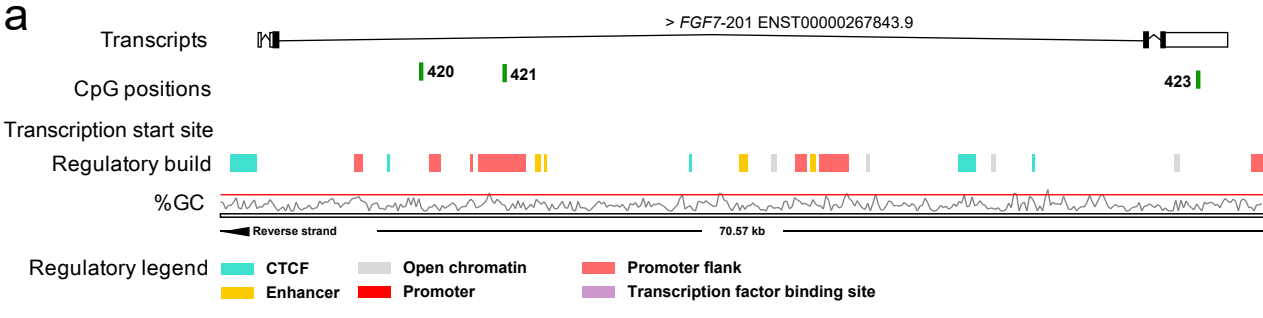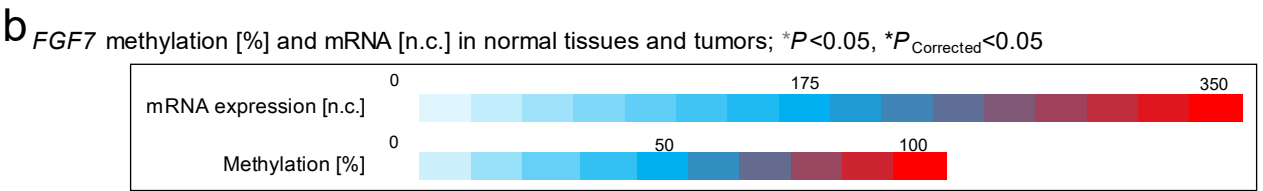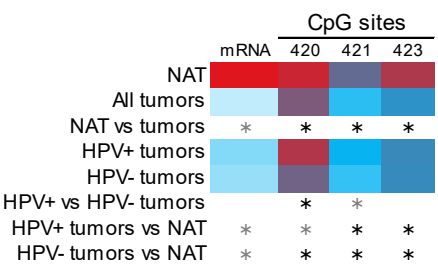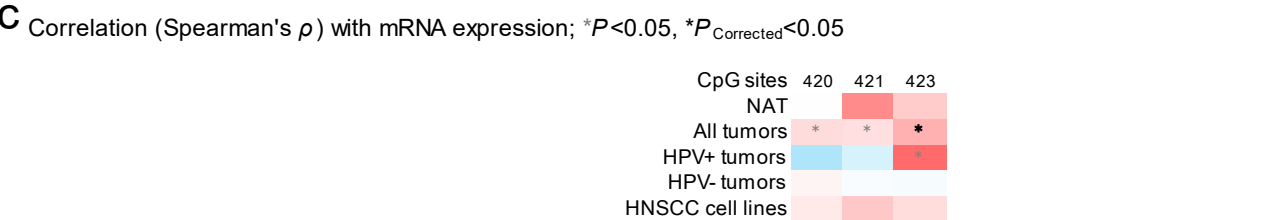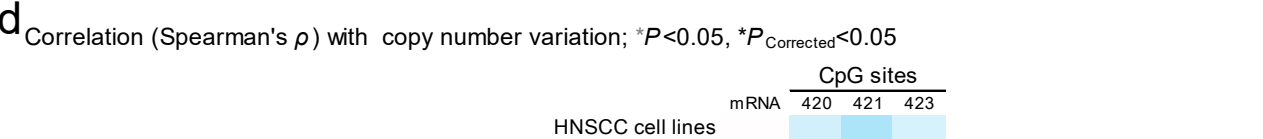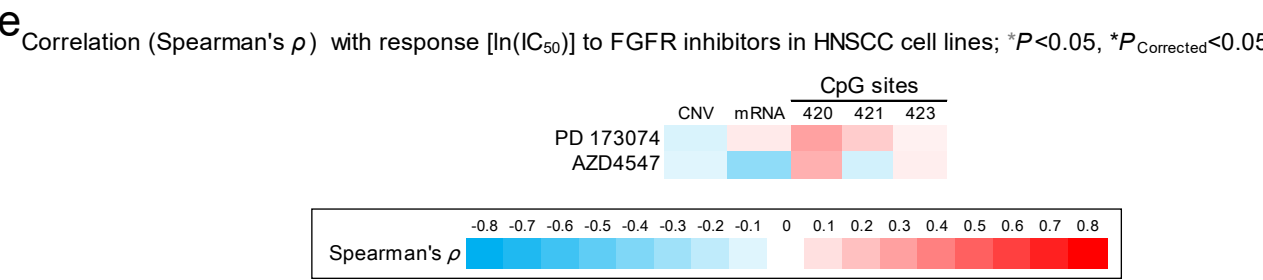

Supplement: Supplementary file 8 — Additional file 8: Fig. S6. This figure illustrates correlation and association of FGF7 DNA methylation with mRNA expression, HPV status, copy number variation, and sensitivity to the FGFR-targeted TKIs PD 173074 and AZD4547. Exemplarily, results of three selected CpG sites within FGF7 are illustrated. [file 13148_2021_1212_MOESM8_ESM.pdf]

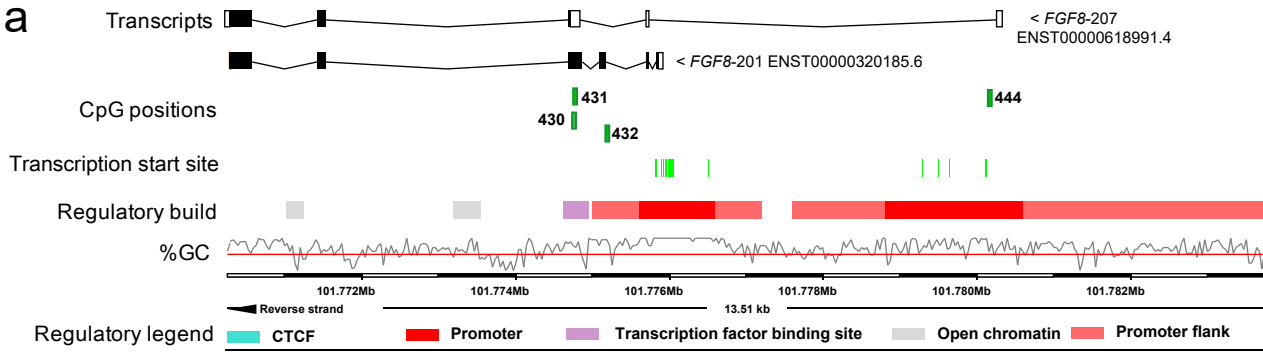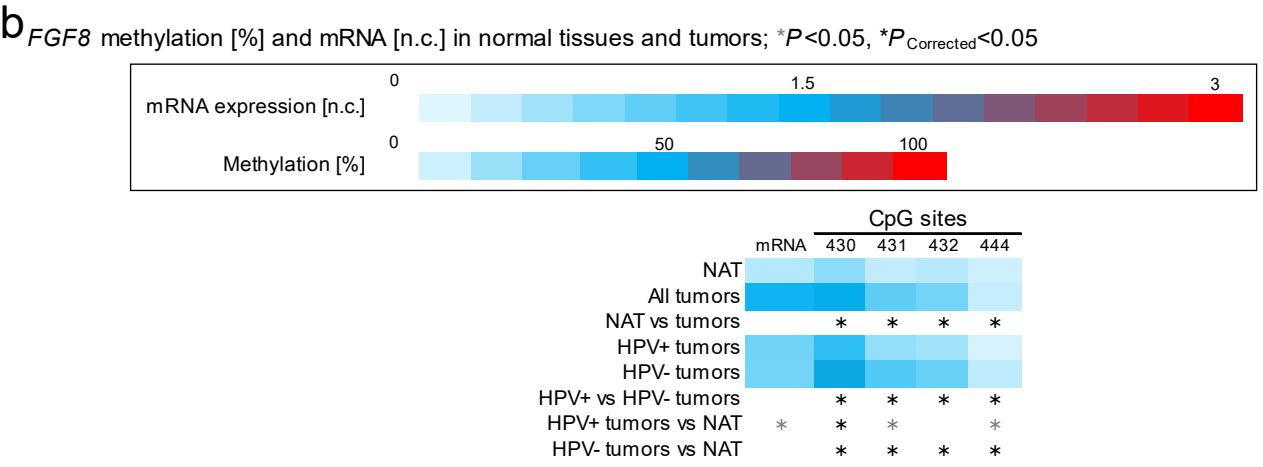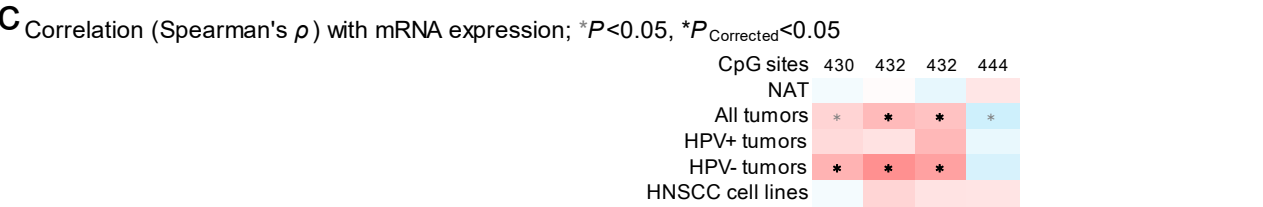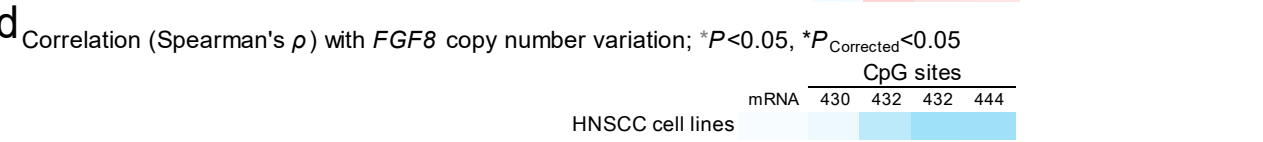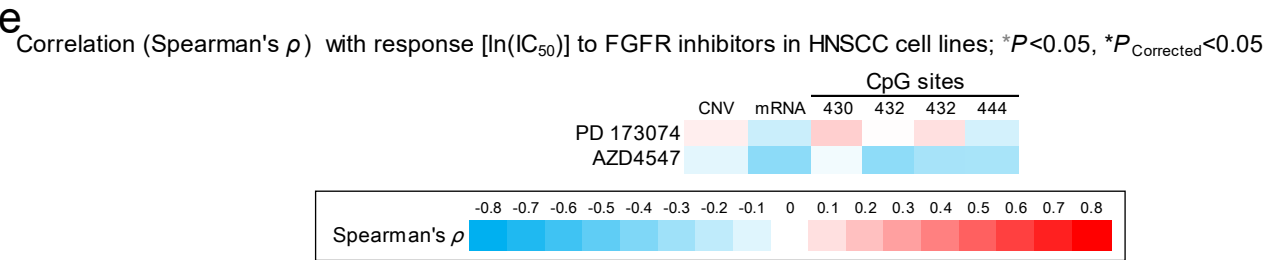

Supplement: Supplementary file 9 — Additional file 9: Fig. S7. This figure illustrates correlation and association of FGF8 DNA methylation with mRNA expression, HPV status, and copy number variation, sensitivity to the FGFR-targeted TKIs PD 173074 and AZD4547. Exemplarily, results of four selected CpG sites within FGF8 are illustrated. [file 13148_2021_1212_MOESM9_ESM.pdf]

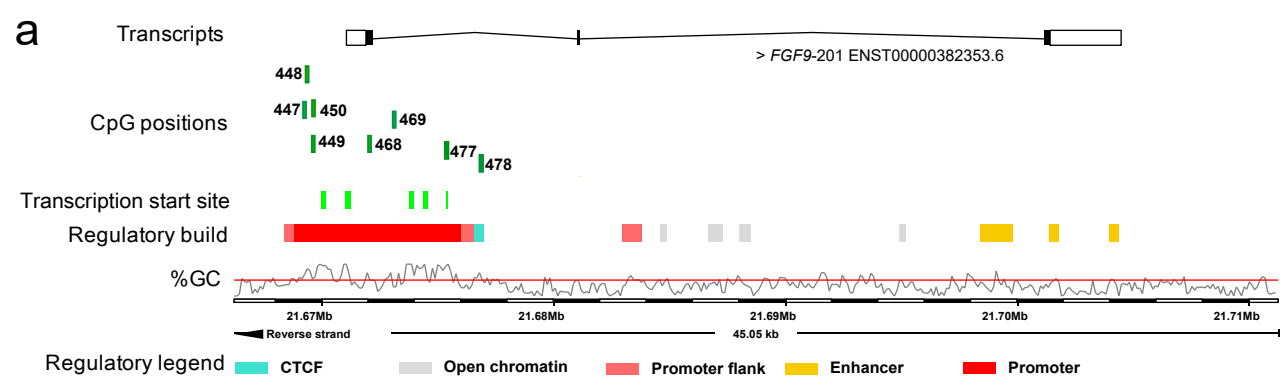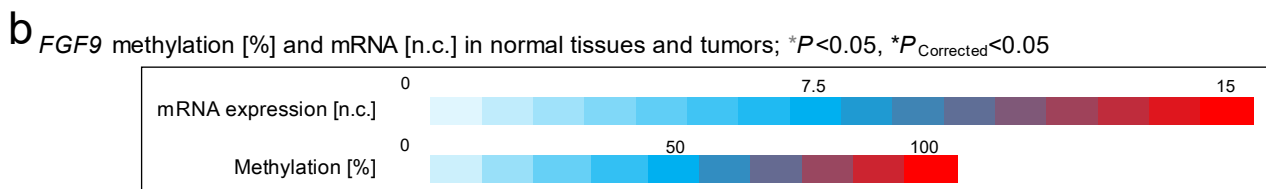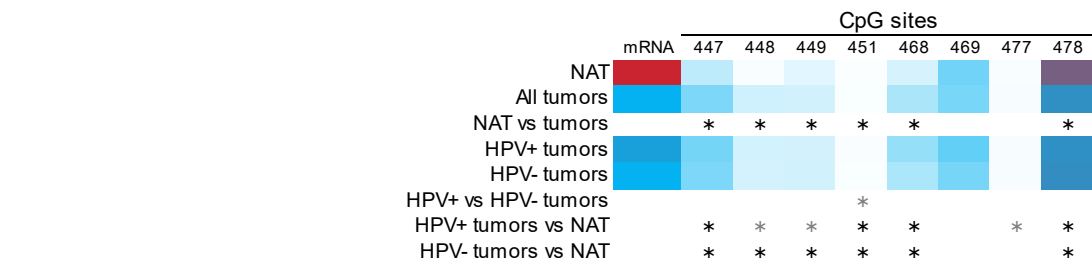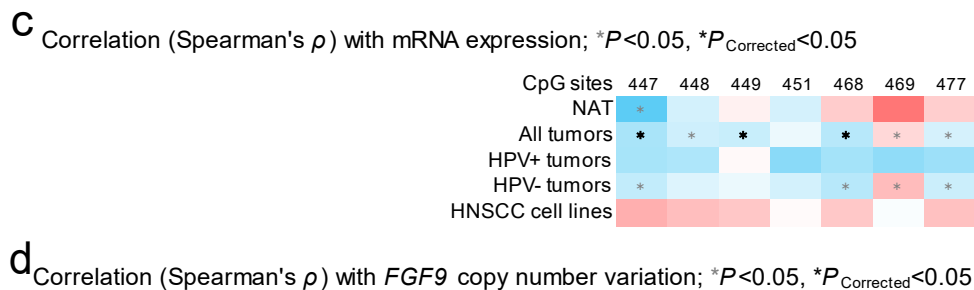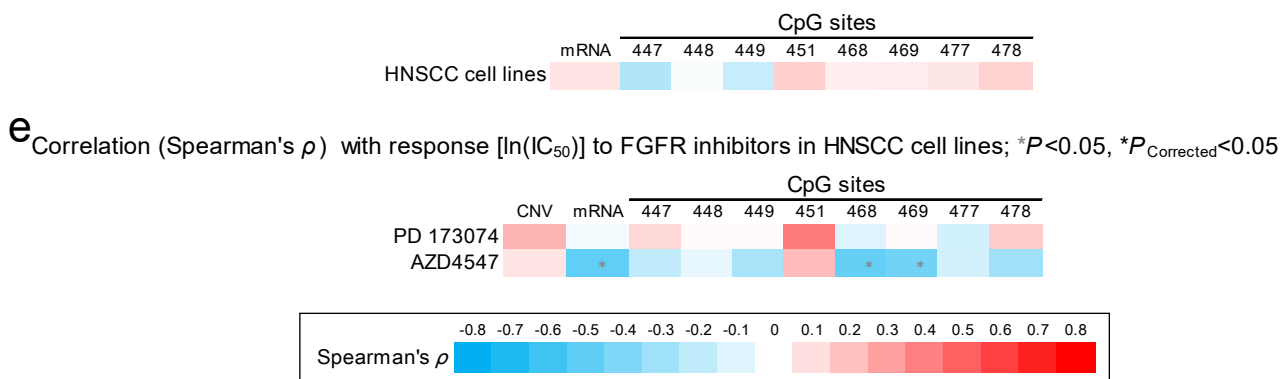

Supplement: Supplementary file 10 — Additional file 10: Fig. S8. This figure illustrates correlation and association of FGF9 DNA methylation with mRNA expression, HPV status, copy number variation, and sensitivity to the FGFR-targeted TKIs PD 173074 and AZD4547. Exemplarily, results of eight selected CpG sites within FGF9 are illustrated. [file 13148_2021_1212_MOESM10_ESM.pdf]

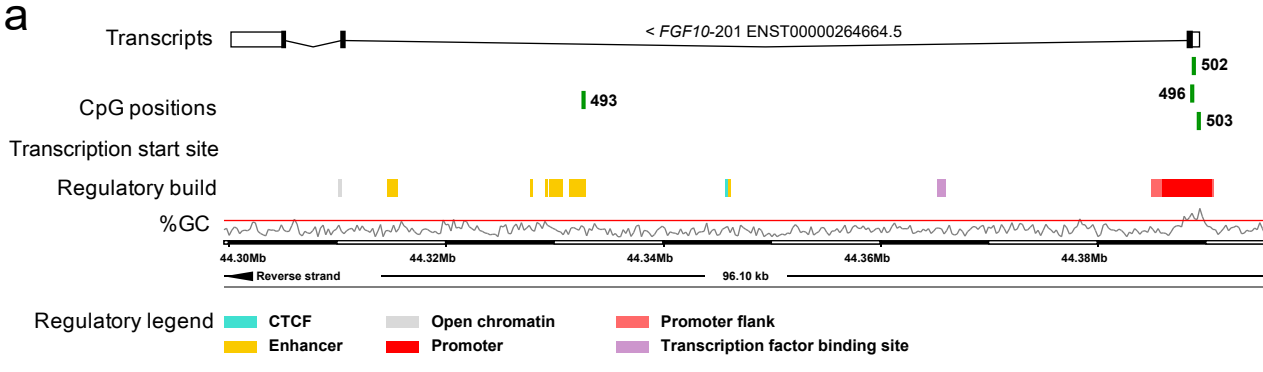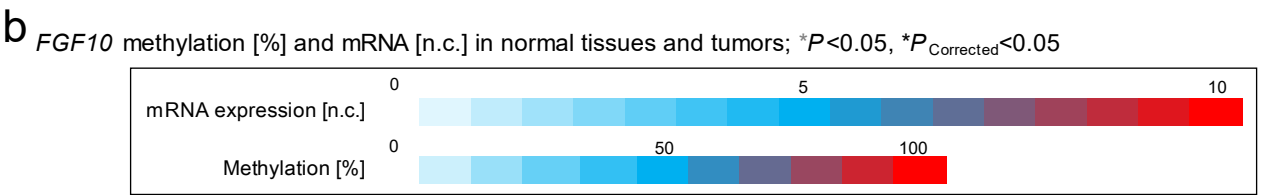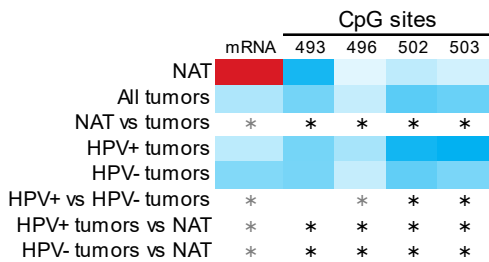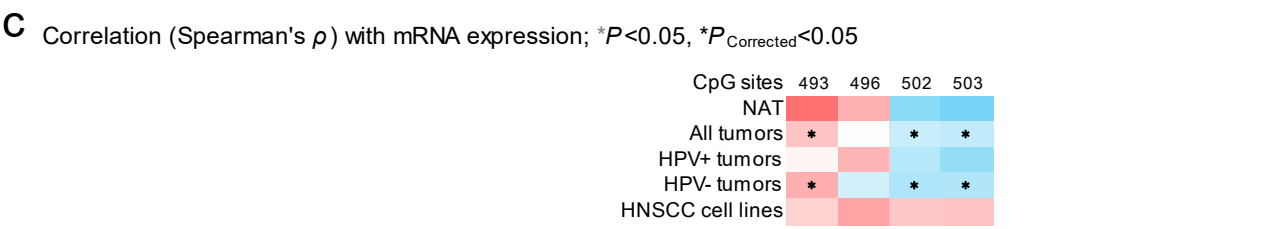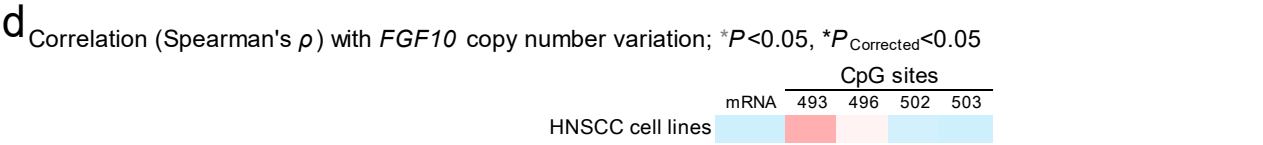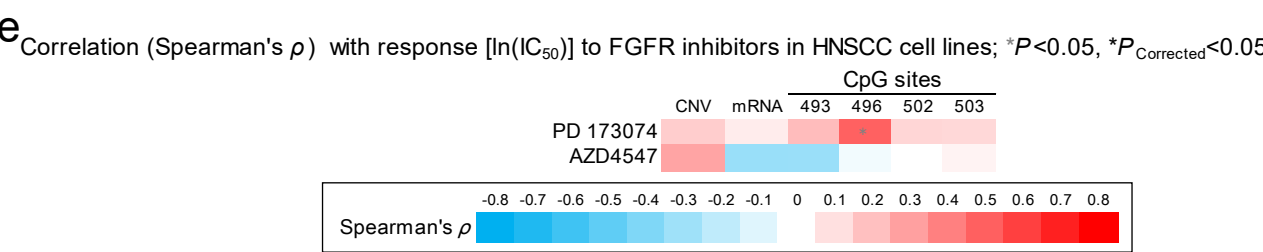

Supplement: Supplementary file 11 — Additional file 11: Fig. S9. This figure illustrates correlation and association of FGF10 DNA methylation with mRNA expression, HPV status, copy number variation, and sensitivity to the FGFR-targeted TKIs PD 173074 and AZD4547. Exemplarily, results of 4 selected CpG sites within FGF10 are illustrated. [file 13148_2021_1212_MOESM11_ESM.pdf]

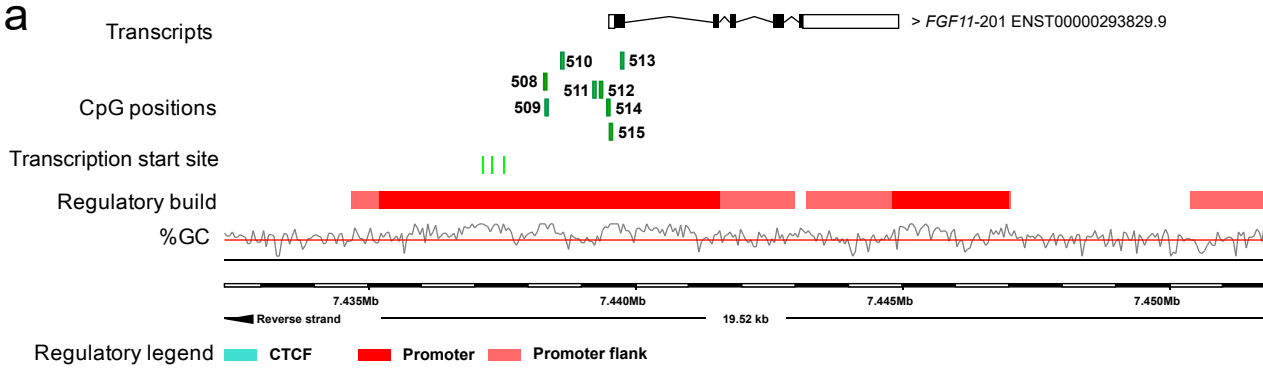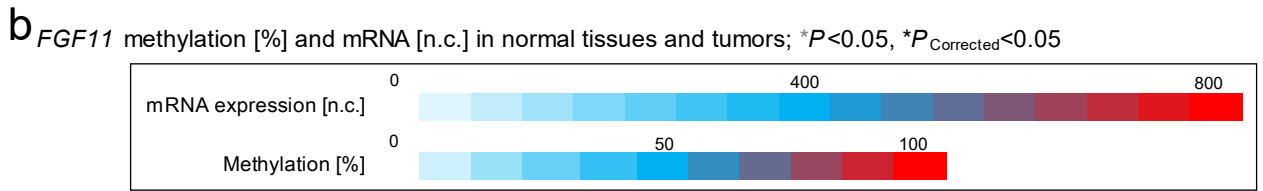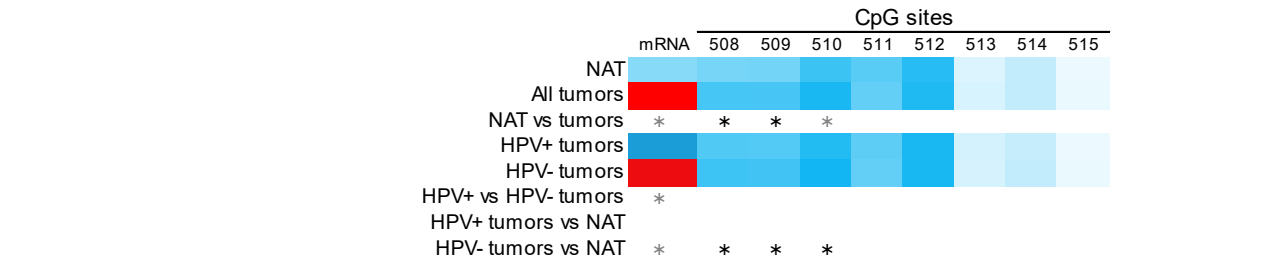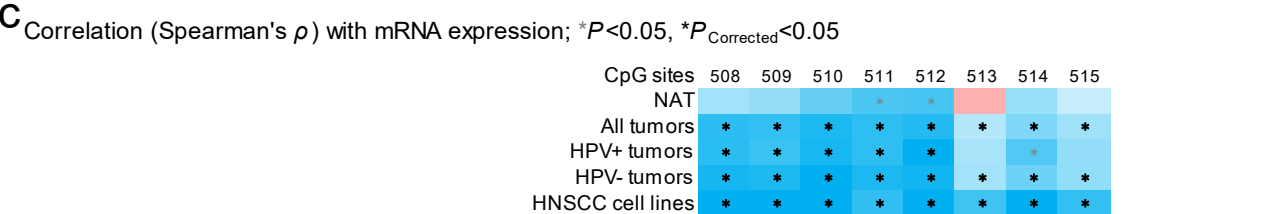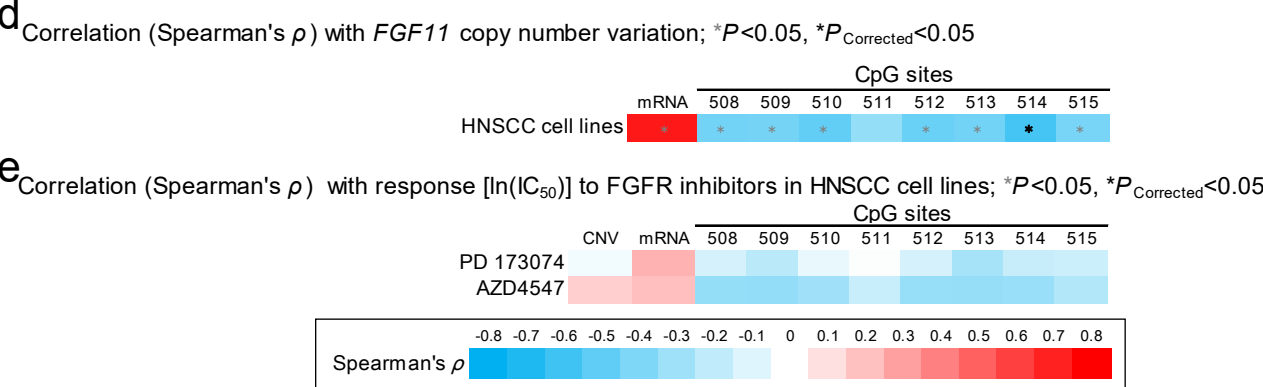

Supplement: Supplementary file 12 — Additional file 12: Fig. S10. This figure illustrates correlation and association of FGF11 DNA methylation with mRNA expression, HPV status, copy number variation, and sensitivity to the FGFR-targeted TKIs PD 173074 and AZD4547. Exemplarily, results of eight selected CpG sites within FGF11 are illustrated. [file 13148_2021_1212_MOESM12_ESM.pdf]

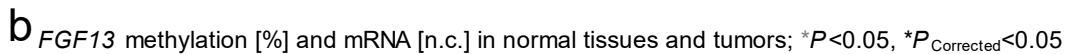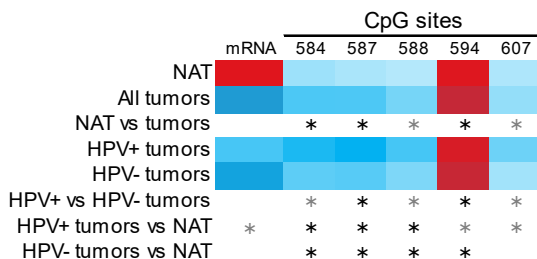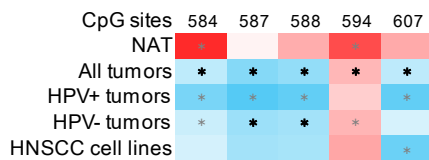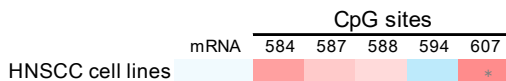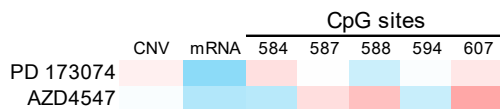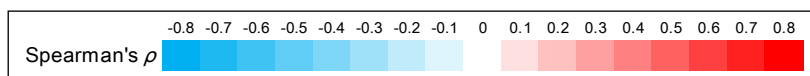

Supplement: Supplementary file 14 — Additional file 14: Fig. S12. This figure illustrates correlation and association of FGF13 DNA methylation with mRNA expression, HPV status, copy number variation, and sensitivity to the FGFR-targeted TKIs PD 173074 and AZD4547. Exemplarily, results of five selected CpG sites within FGF13 are illustrated. [file 13148_2021_1212_MOESM14_ESM.pdf]

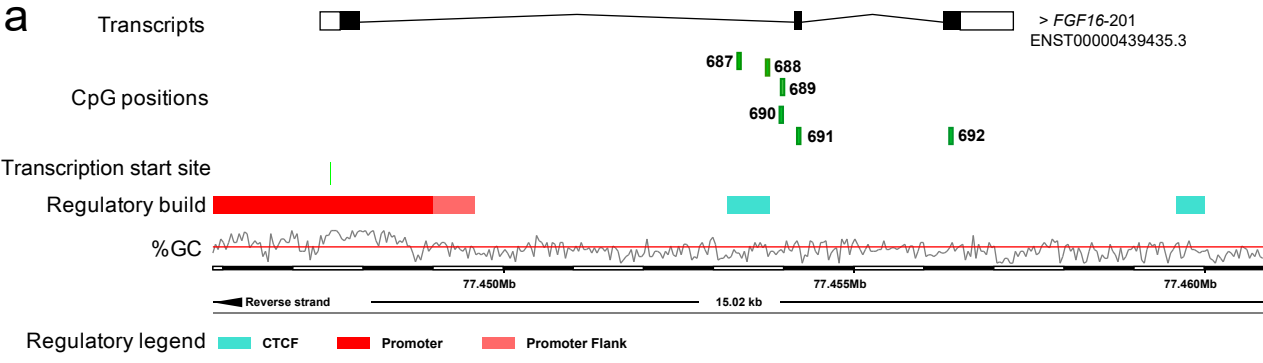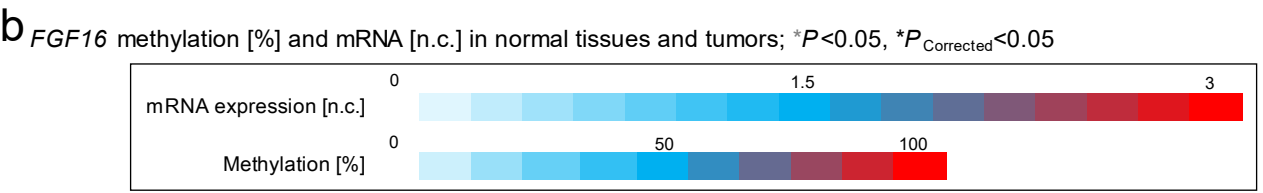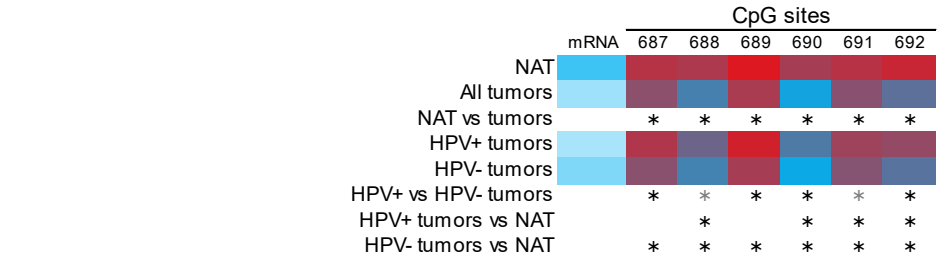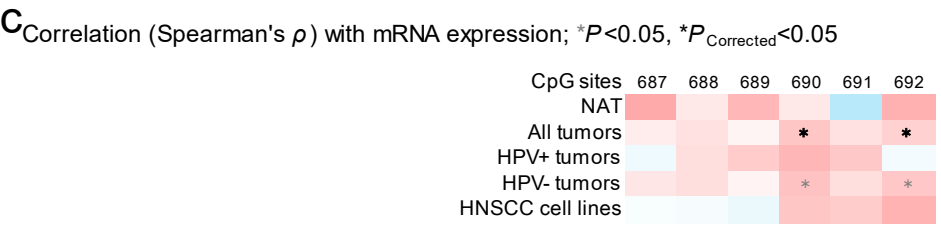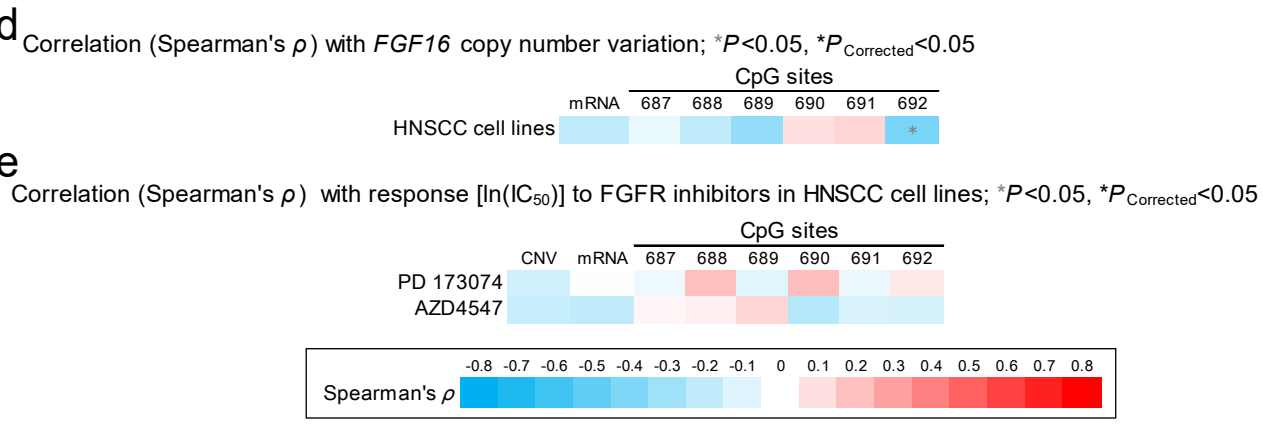

Supplement: Supplementary file 16 — Additional file 16: Fig. S14. This figure illustrates correlation and association of FGF16 DNA methylation with mRNA expression, HPV status, copy number variation, and sensitivity to the FGFR-targeted TKIs PD 173074 and AZD4547. Exemplarily, results of six selected CpG sites within FGF16 are illustrated. [file 13148_2021_1212_MOESM16_ESM.pdf]

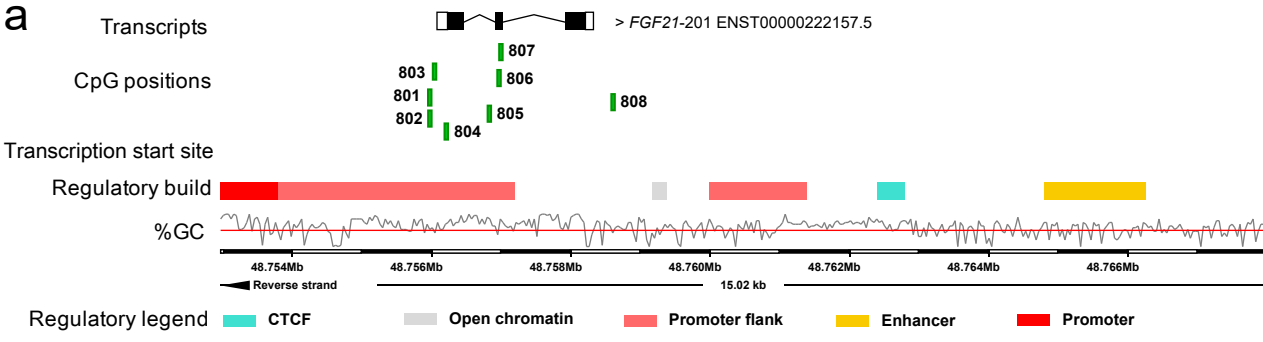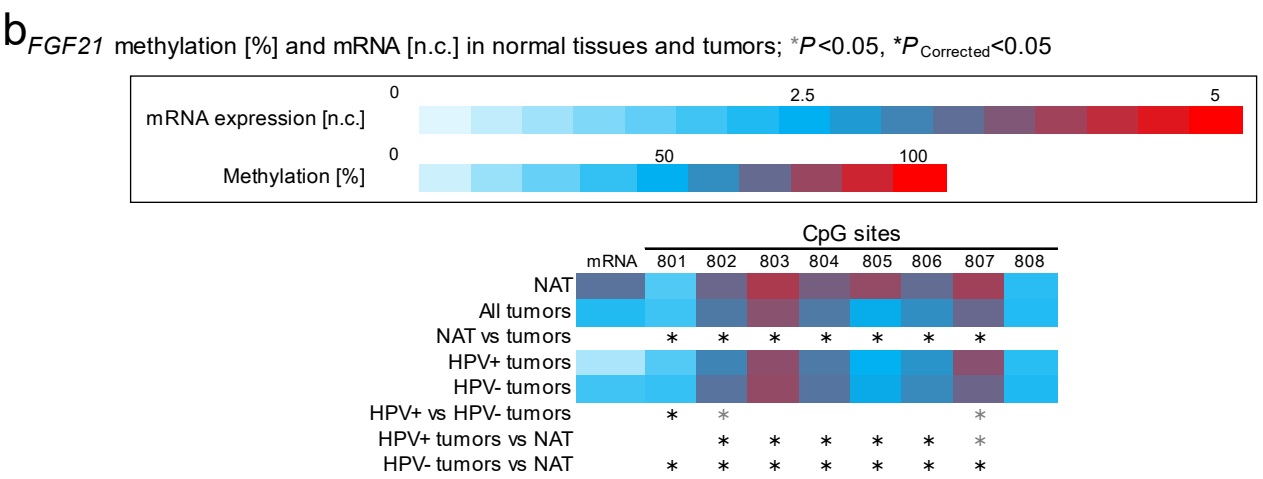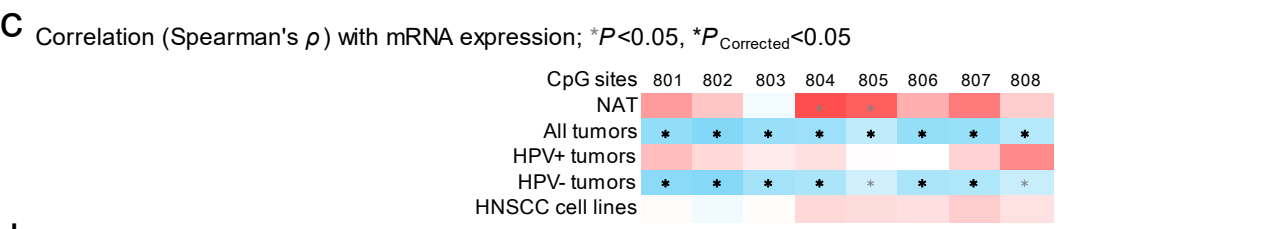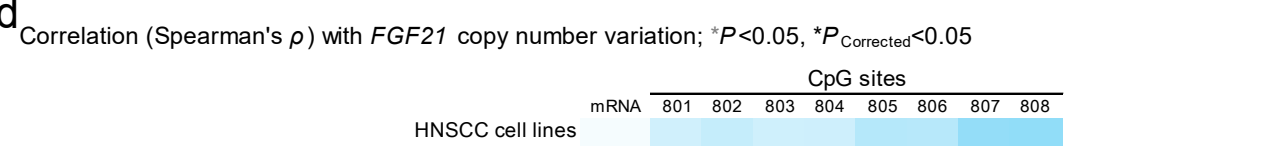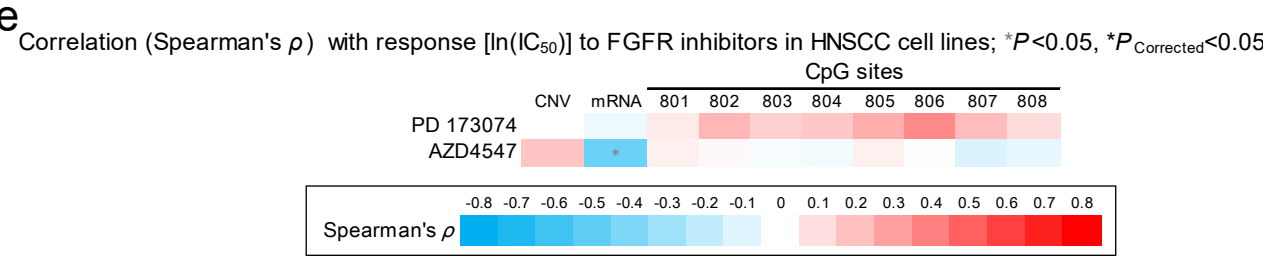

Supplement: Supplementary file 20 — Additional file 20: Fig. S18. This figure illustrates correlation and association of FGF21 DNA methylation with mRNA expression, HPV status, copy number variation, and sensitivity to the FGFR-targeted TKIs PD 173074 and AZD4547. Exemplarily, results of eight selected CpG sites within FGF21 are illustrated. [file 13148_2021_1212_MOESM20_ESM.pdf]
