# Supplementary material for: Fibroblast growth factor (FGF), FGF receptor (FGFR), and cyclin D1 (CCND1) DNA methylation in head and neck squamous cell carcinomas is associated with transcriptional activity, gene amplification, human papillomavirus (HPV) status, and sensitivity to tyrosine kinase inhibitors
Source: Clin Epigenetics. 2021 Dec 21;13:228. doi: 10.1186/s13148-021-01212-4 (PMC8693503; doi:10.1186/s13148-021-01212-4)
Supplement: Supplementary file 7 — Additional file 7: Fig. S5. This figure illustrates correlation and association of FGF23 and FGF6 DNA methylation with mRNA expression, HPV status, copy number variation, and sensitivity to the FGFR-targeted TKIs PD 173074 and AZD4547. Exemplarily, results of five selected CpG sites within FGF23 and FGF6 are illustrated. [file 13148_2021_1212_MOESM7_ESM.pdf]

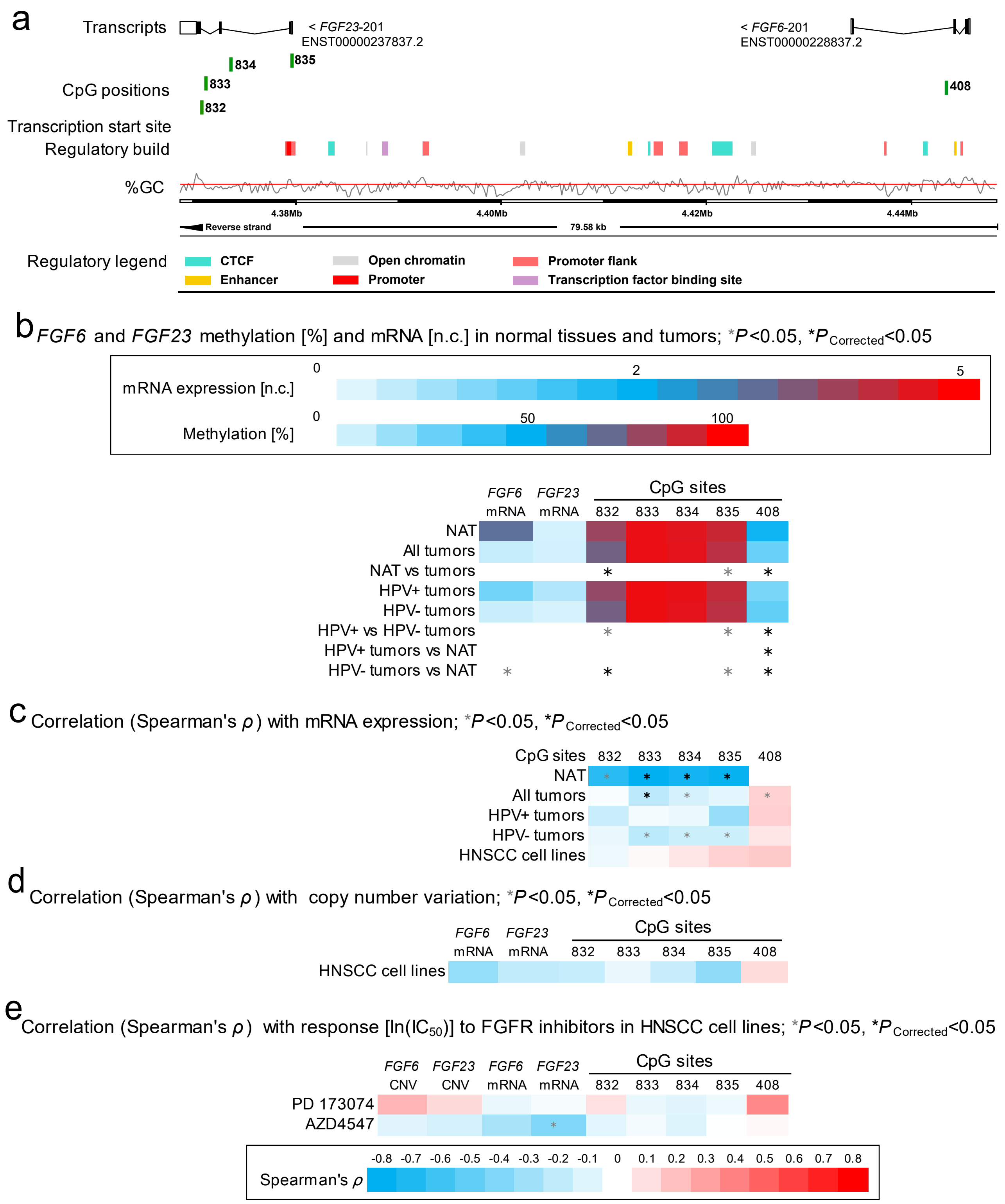
a
Transcripts
< FGF23-201
ENST00000237837.2
< FGF6-201
ENST00000228837.2
CpG positions
834
835
833
832
408
Transcription start site
Regulatory build
%GC
4.38Mb
4.40Mb
4.42Mb
4.44Mb
Reverse strand
79.58 kb
Regulatory legend
CTCF
Enhancer
Open chromatin
Promoter
Promoter flank
Transcription factor binding site
b
FGF6 and FGF23 methylation [%] and mRNA [n.c.] in normal tissues and tumors; *P<0.05, *PCorrected<0.05
mRNA expression [n.c.]
0
2
5
Methylation [%]
0
50
100
FGF6 mRNA
FGF23 mRNA
CpG sites
832
833
834
835
408
NAT
All tumors
NAT vs tumors
HPV+ tumors
HPV- tumors
HPV+ vs HPV- tumors
HPV+ tumors vs NAT
HPV- tumors vs NAT
c
Correlation (Spearman's ρ) with mRNA expression; *P<0.05, *PCorrected<0.05
CpG sites
832
833
834
835
408
NAT
All tumors
HPV+ tumors
HPV- tumors
HNSCC cell lines
d
Correlation (Spearman's ρ) with copy number variation; *P<0.05, *PCorrected<0.05
FGF6 mRNA
FGF23 mRNA
CpG sites
832
833
834
835
408
HNSCC cell lines
e
Correlation (Spearman's ρ) with response [ln(IC50)] to FGFR inhibitors in HNSCC cell lines; *P<0.05, *PCorrected<0.05
FGF6 CNV
FGF23 CNV
FGF6 mRNA
FGF23 mRNA
CpG sites
832
833
834
835
408
PD 173074
AZD4547
Spearman's ρ
-0.8
-0.7
-0.6
-0.5
-0.4
-0.3
-0.2
-0.1
0
0.1
0.2
0.3
0.4
0.5
0.6
0.7
0.8
