# Supplementary material for: Fibroblast growth factor (FGF), FGF receptor (FGFR), and cyclin D1 (CCND1) DNA methylation in head and neck squamous cell carcinomas is associated with transcriptional activity, gene amplification, human papillomavirus (HPV) status, and sensitivity to tyrosine kinase inhibitors
Source: Clin Epigenetics. 2021 Dec 21;13:228. doi: 10.1186/s13148-021-01212-4 (PMC8693503; doi:10.1186/s13148-021-01212-4)
Supplement: Supplementary file 13 — Additional file 13: Fig. S11. This figure illustrates correlation and association of FGF12 DNA methylation with mRNA expression, HPV status, copy number variation, and sensitivity to the FGFR-targeted TKIs PD 173074 and AZD4547. Exemplarily, results of 12 selected CpG sites within FGF12 are illustrated. [file 13148_2021_1212_MOESM13_ESM.pdf]

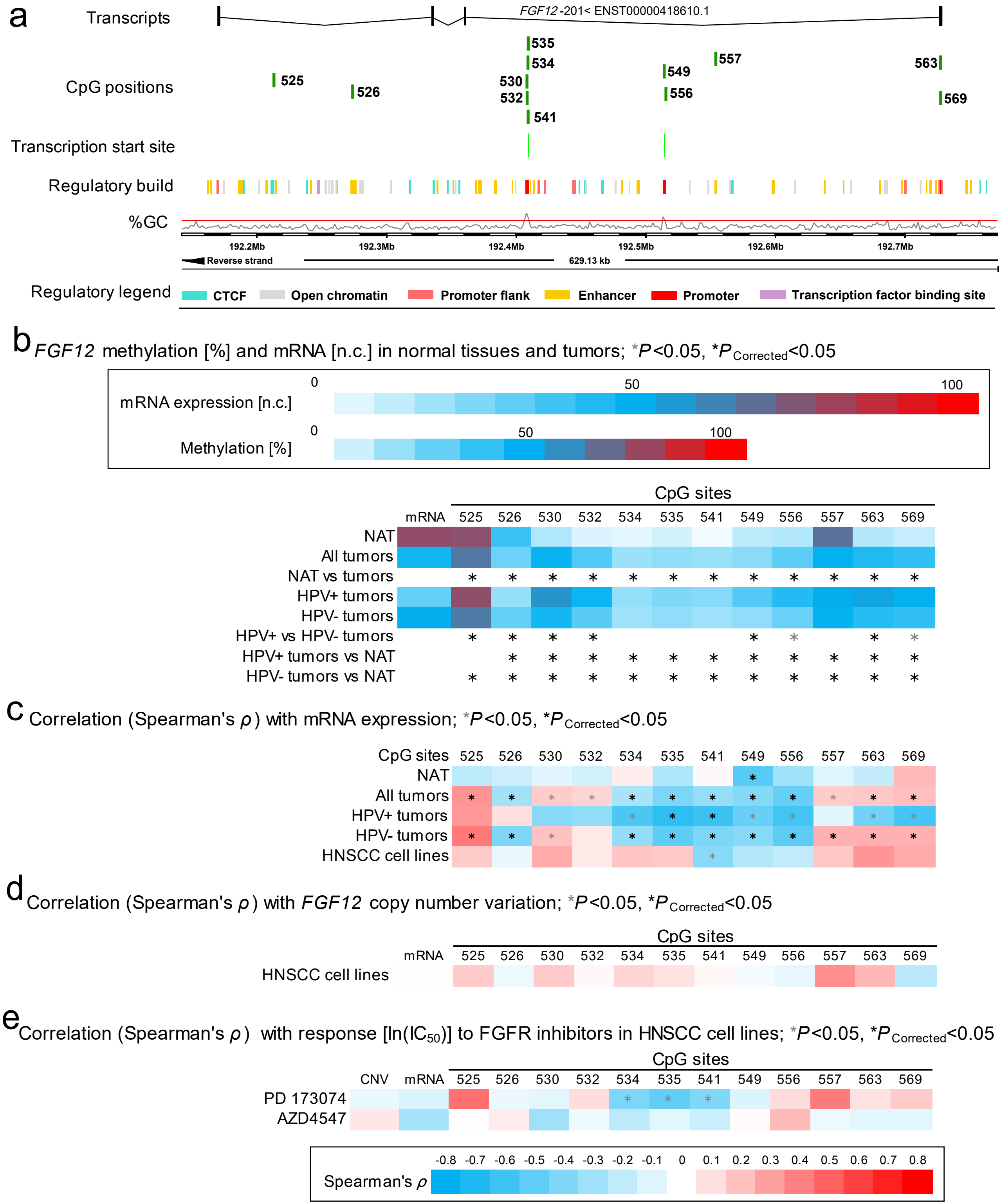
a
Transcripts
FGF12-201< ENST00000418610.1
CpG positions
525
526
530
532
535
534
541
549
556
557
563
569
Transcription start site
Regulatory build
%GC
192.2Mb
192.3Mb
192.4Mb
192.5Mb
192.6Mb
192.7Mb
Reverse strand
629.13 kb
Regulatory legend
CTCF
Open chromatin
Promoter flank
Enhancer
Promoter
Transcription factor binding site
b FGF12 methylation [%] and mRNA [n.c.] in normal tissues and tumors; *P<0.05, *P Corrected<0.05
mRNA expression [n.c.]
0
50
100
Methylation [%]
0
50
100
CpG sites
mRNA 525 526 530 532 534 535 541 549 556 557 563 569
NAT
All tumors
NAT vs tumors
HPV+ tumors
HPV- tumors
HPV+ vs HPV- tumors
HPV+ tumors vs NAT
HPV- tumors vs NAT
c Correlation (Spearman's ρ) with mRNA expression; *P<0.05, *P Corrected<0.05
CpG sites 525 526 530 532 534 535 541 549 556 557 563 569
NAT
All tumors
HPV+ tumors
HPV- tumors
HNSCC cell lines
d Correlation (Spearman's ρ) with FGF12 copy number variation; *P<0.05, *P Corrected<0.05
CpG sites
mRNA 525 526 530 532 534 535 541 549 556 557 563 569
HNSCC cell lines
e Correlation (Spearman's ρ) with response [ln(IC50)] to FGFR inhibitors in HNSCC cell lines; *P<0.05, *P Corrected<0.05
CpG sites
CNV mRNA 525 526 530 532 534 535 541 549 556 557 563 569
PD 173074
AZD4547
Spearman's ρ
-0.8 -0.7 -0.6 -0.5 -0.4 -0.3 -0.2 -0.1 0 0.1 0.2 0.3 0.4 0.5 0.6 0.7 0.8
