# Supplementary material for: Fibroblast growth factor (FGF), FGF receptor (FGFR), and cyclin D1 (CCND1) DNA methylation in head and neck squamous cell carcinomas is associated with transcriptional activity, gene amplification, human papillomavirus (HPV) status, and sensitivity to tyrosine kinase inhibitors
Source: Clin Epigenetics. 2021 Dec 21;13:228. doi: 10.1186/s13148-021-01212-4 (PMC8693503; doi:10.1186/s13148-021-01212-4)
Supplement: Supplementary file 15 — Additional file 15: Fig. S13. This figure illustrates correlation and association of FGF14 DNA methylation with mRNA expression, HPV status, copy number variation, and sensitivity to the FGFR-targeted TKIs PD 173074 and AZD4547. Exemplarily, results of ten selected CpG sites within FGF14 are illustrated. [file 13148_2021_1212_MOESM15_ESM.pdf]

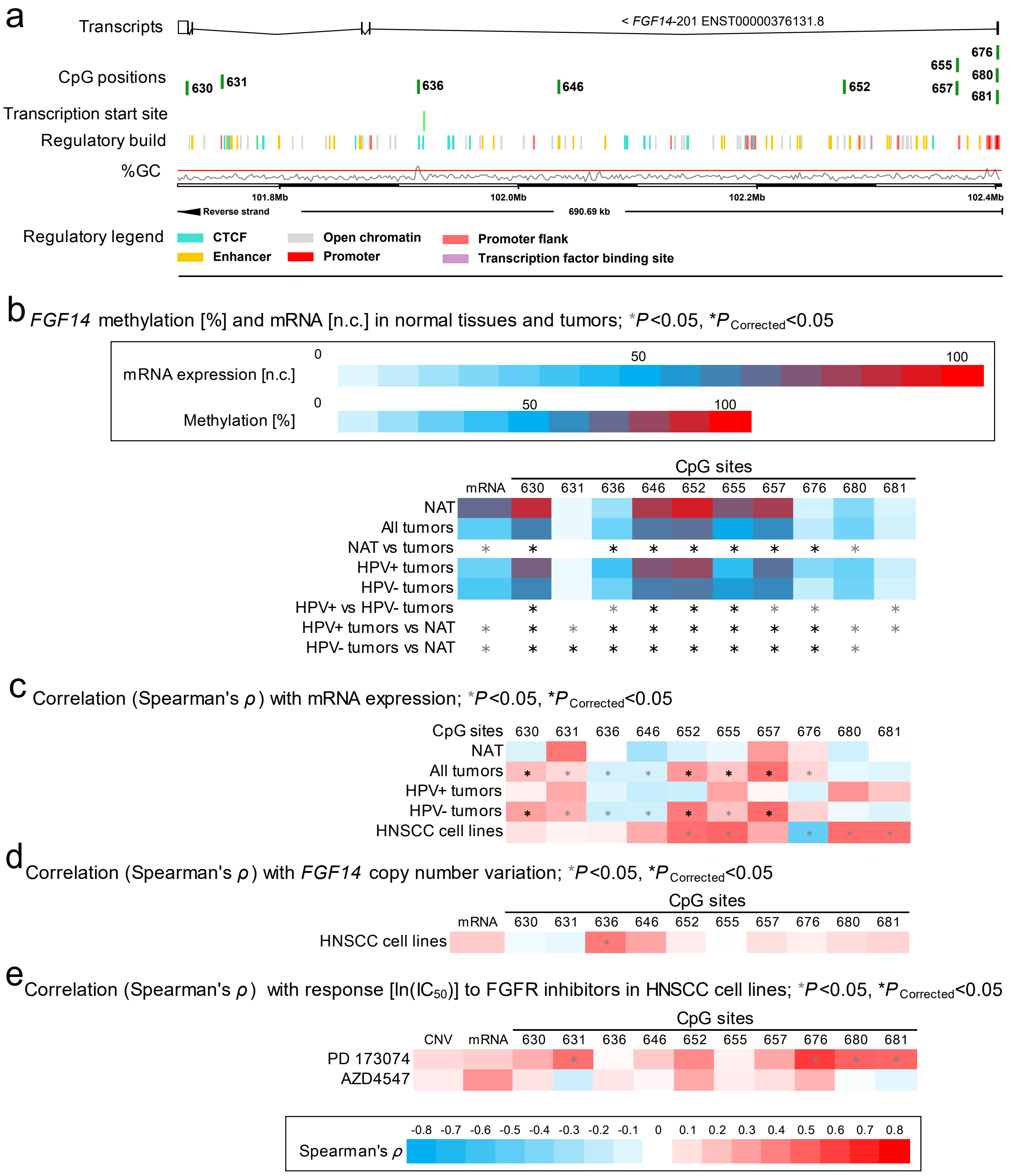
a
Transcripts
< FGF14-201 ENST00000376131.8
CpG positions
630
631
636
646
652
655
657
676
680
681
Transcription start site
Regulatory build
%GC
101.8Mb
102.0Mb
102.2Mb
102.4Mb
Reverse strand
690.69 kb
Regulatory legend
CTCF
Enhancer
Open chromatin
Promoter
Promoter flank
Transcription factor binding site
b FGF14 methylation [%] and mRNA [n.c.] in normal tissues and tumors; *P<0.05, *P Corrected<0.05
mRNA expression [n.c.]
0
50
100
Methylation [%]
0
50
100
CpG sites
mRNA 630 631 636 646 652 655 657 676 680 681
NAT
All tumors
NAT vs tumors
HPV+ tumors
HPV- tumors
HPV+ vs HPV- tumors
HPV+ tumors vs NAT
HPV- tumors vs NAT
c Correlation (Spearman's ρ) with mRNA expression; *P<0.05, *P Corrected<0.05
CpG sites 630 631 636 646 652 655 657 676 680 681
NAT
All tumors
HPV+ tumors
HPV- tumors
HNSCC cell lines
d Correlation (Spearman's ρ) with FGF14 copy number variation; *P<0.05, *P Corrected<0.05
CpG sites
mRNA 630 631 636 646 652 655 657 676 680 681
HNSCC cell lines
e Correlation (Spearman's ρ) with response [ln(IC50)] to FGFR inhibitors in HNSCC cell lines; *P<0.05, *P Corrected<0.05
CpG sites
CNV mRNA 630 631 636 646 652 655 657 676 680 681
PD 173074
AZD4547
Spearman's ρ
-0.8 -0.7 -0.6 -0.5 -0.4 -0.3 -0.2 -0.1 0 0.1 0.2 0.3 0.4 0.5 0.6 0.7 0.8
