# Supplementary material for: Fibroblast growth factor (FGF), FGF receptor (FGFR), and cyclin D1 (CCND1) DNA methylation in head and neck squamous cell carcinomas is associated with transcriptional activity, gene amplification, human papillomavirus (HPV) status, and sensitivity to tyrosine kinase inhibitors
Source: Clin Epigenetics. 2021 Dec 21;13:228. doi: 10.1186/s13148-021-01212-4 (PMC8693503; doi:10.1186/s13148-021-01212-4)
Supplement: Supplementary file 17 — Additional file 17: Fig. S15. This figure illustrates correlation and association of FGF17 DNA methylation with mRNA expression, HPV status, copy number variation, and sensitivity to the FGFR-targeted TKIs PD 173074 and AZD4547. Exemplarily, results of seven selected CpG sites within FGF17 are illustrated. [file 13148_2021_1212_MOESM17_ESM.pdf]

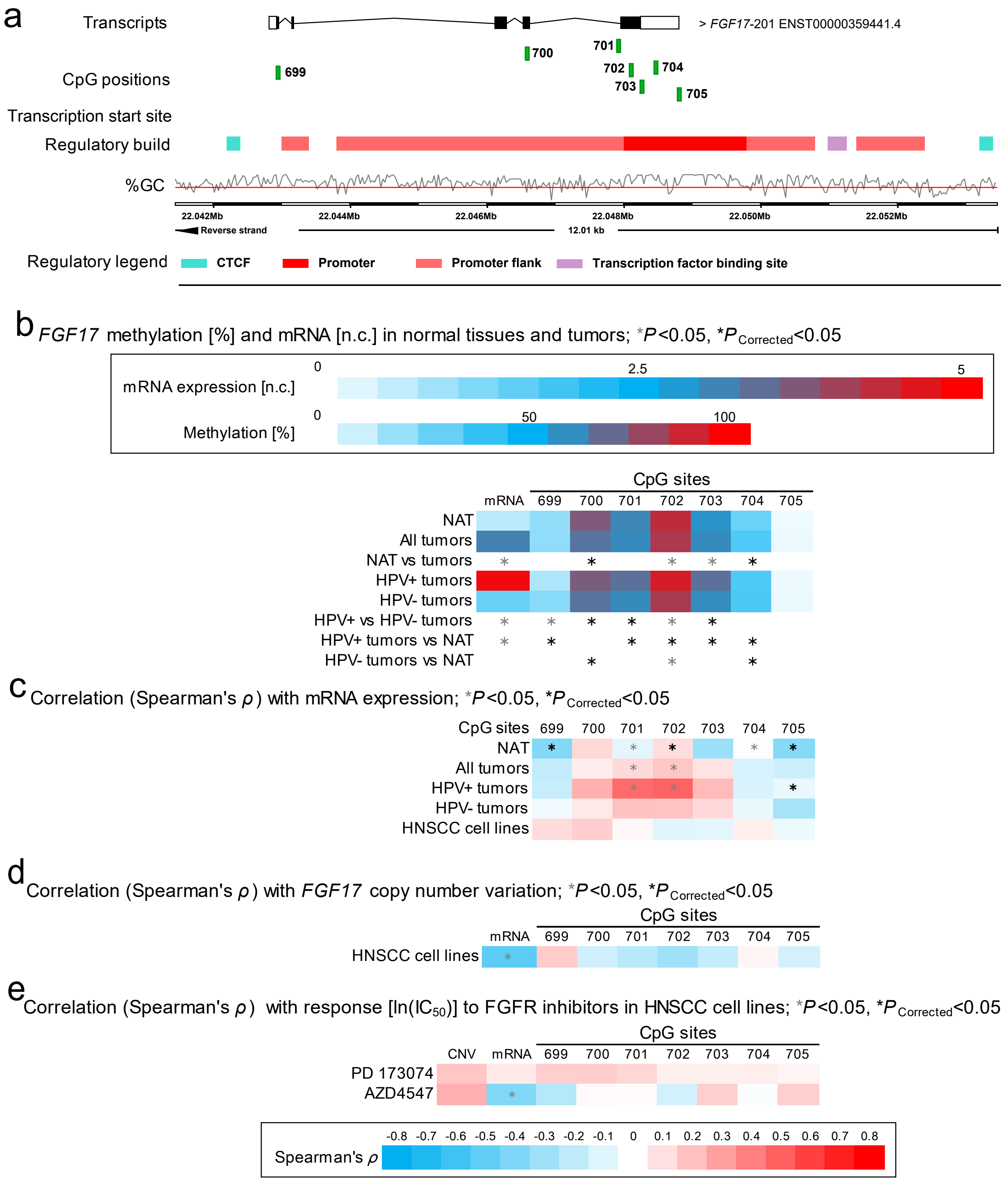
a
Transcripts
> FGF17-201 ENST00000359441.4
CpG positions
699
700
701
702
703
704
705
Transcription start site
Regulatory build
%GC
22.042Mb
22.044Mb
22.046Mb
22.048Mb
22.050Mb
22.052Mb
Reverse strand
12.01 kb
Regulatory legend
CTCF
Promoter
Promoter flank
Transcription factor binding site
b
FGF17 methylation [%] and mRNA [n.c.] in normal tissues and tumors; *P<0.05, *PCorrected<0.05
mRNA expression [n.c.]
0
2.5
5
Methylation [%]
0
50
100
CpG sites
mRNA 699 700 701 702 703 704 705
NAT
All tumors
NAT vs tumors
HPV+ tumors
HPV- tumors
HPV+ vs HPV- tumors
HPV+ tumors vs NAT
HPV- tumors vs NAT
c
Correlation (Spearman's ρ) with mRNA expression; *P<0.05, *PCorrected<0.05
CpG sites 699 700 701 702 703 704 705
NAT
All tumors
HPV+ tumors
HPV- tumors
HNSCC cell lines
d
Correlation (Spearman's ρ) with FGF17 copy number variation; *P<0.05, *PCorrected<0.05
CpG sites
mRNA 699 700 701 702 703 704 705
HNSCC cell lines
e
Correlation (Spearman's ρ) with response [ln(IC50)] to FGFR inhibitors in HNSCC cell lines; *P<0.05, *PCorrected<0.05
CpG sites
CNV mRNA 699 700 701 702 703 704 705
PD 173074
AZD4547
Spearman's ρ
-0.8 -0.7 -0.6 -0.5 -0.4 -0.3 -0.2 -0.1 0 0.1 0.2 0.3 0.4 0.5 0.6 0.7 0.8
