# Supplementary material for: Fibroblast growth factor (FGF), FGF receptor (FGFR), and cyclin D1 (CCND1) DNA methylation in head and neck squamous cell carcinomas is associated with transcriptional activity, gene amplification, human papillomavirus (HPV) status, and sensitivity to tyrosine kinase inhibitors
Source: Clin Epigenetics. 2021 Dec 21;13:228. doi: 10.1186/s13148-021-01212-4 (PMC8693503; doi:10.1186/s13148-021-01212-4)
Supplement: Supplementary file 18 — Additional file 18: Fig. S16. This figure illustrates correlation and association of FGF18 DNA methylation with mRNA expression, HPV status, copy number variation, and sensitivity to the FGFR-targeted TKIs PD 173074 and AZD4547. Exemplarily, results of 12 selected CpG sites within FGF18 are illustrated. [file 13148_2021_1212_MOESM18_ESM.pdf]

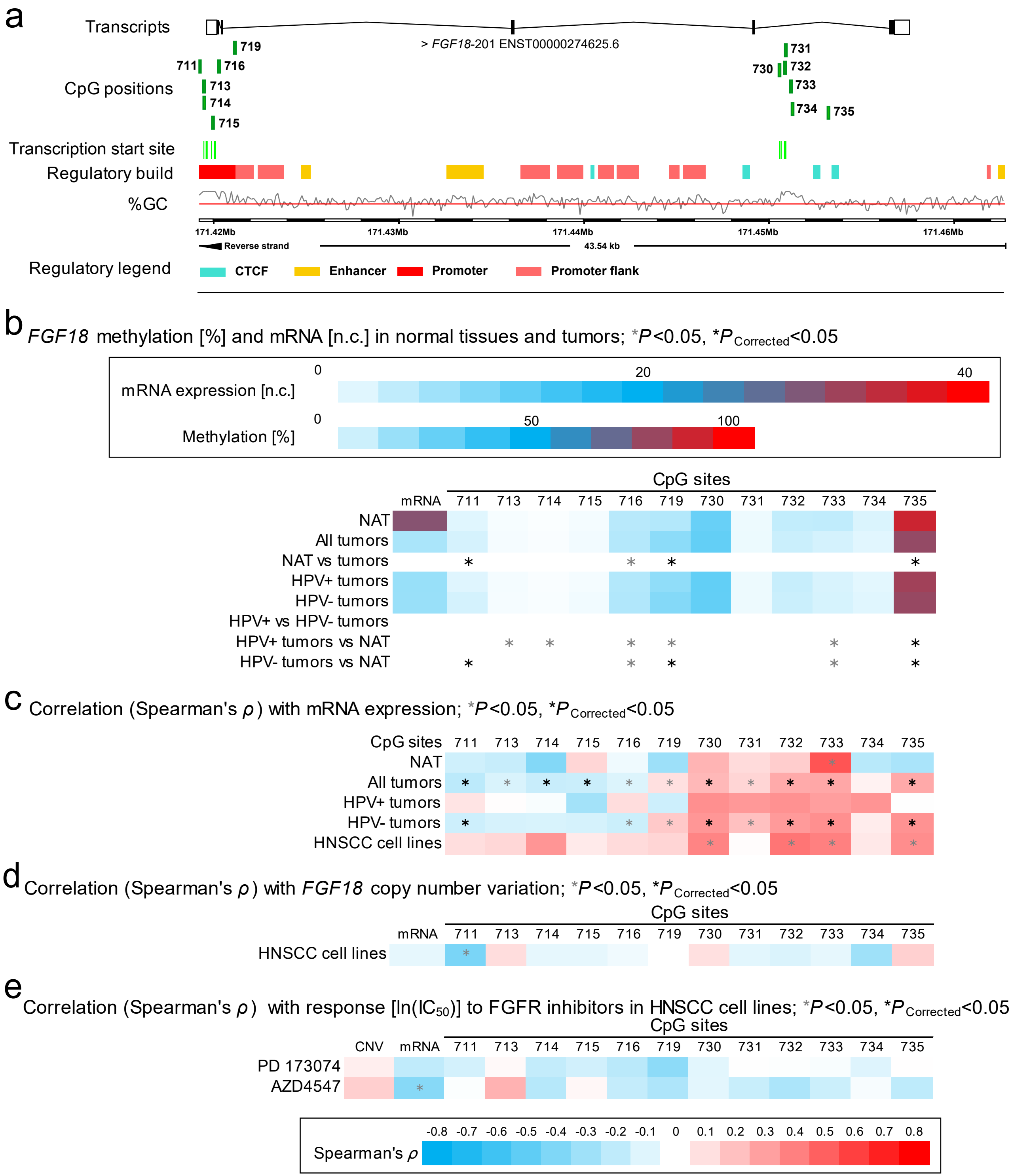

a
Transcripts
> FGF18-201 ENST00000274625.6
CpG positions
719
711
716
713
714
715
731
730
732
733
734
735
Transcription start site
Regulatory build
%GC
171.42Mb
171.43Mb
171.44Mb
171.45Mb
171.46Mb
Reverse strand
43.54 kb
Regulatory legend
CTCF
Enhancer
Promoter
Promoter flank
b
FGF18 methylation [%] and mRNA [n.c.] in normal tissues and tumors; *P<0.05, *P Corrected<0.05
mRNA expression [n.c.]
0
20
40
Methylation [%]
0
50
100
CpG sites
mRNA
711
713
714
715
716
719
730
731
732
733
734
735
NAT
All tumors
NAT vs tumors
HPV+ tumors
HPV- tumors
HPV+ vs HPV- tumors
HPV+ tumors vs NAT
HPV- tumors vs NAT
c
Correlation (Spearman's ρ) with mRNA expression; *P<0.05, *P Corrected<0.05
CpG sites
NAT
All tumors
HPV+ tumors
HPV- tumors
HNSCC cell lines
d
Correlation (Spearman's ρ) with FGF18 copy number variation; *P<0.05, *P Corrected<0.05
CpG sites
mRNA
HNSCC cell lines
e
Correlation (Spearman's ρ) with response [ln(IC50)] to FGFR inhibitors in HNSCC cell lines; *P<0.05, *P Corrected<0.05
CpG sites
CNV
mRNA
PD 173074
AZD4547
Spearman's ρ
-0.8
-0.7
-0.6
-0.5
-0.4
-0.3
-0.2
-0.1
0
0.1
0.2
0.3
0.4
0.5
0.6
0.7
0.8
