# Supplementary material for: Fibroblast growth factor (FGF), FGF receptor (FGFR), and cyclin D1 (CCND1) DNA methylation in head and neck squamous cell carcinomas is associated with transcriptional activity, gene amplification, human papillomavirus (HPV) status, and sensitivity to tyrosine kinase inhibitors
Source: Clin Epigenetics. 2021 Dec 21;13:228. doi: 10.1186/s13148-021-01212-4 (PMC8693503; doi:10.1186/s13148-021-01212-4)
Supplement: Supplementary file 21 — Additional file 21: Fig. S19. This figure illustrates correlation and association of FGF22 DNA methylation with mRNA expression, HPV status, copy number variation, and sensitivity to the FGFR-targeted TKIs PD 173074 and AZD4547. Exemplarily, results of eight selected CpG sites within FGF22 are illustrated. [file 13148_2021_1212_MOESM21_ESM.pdf]

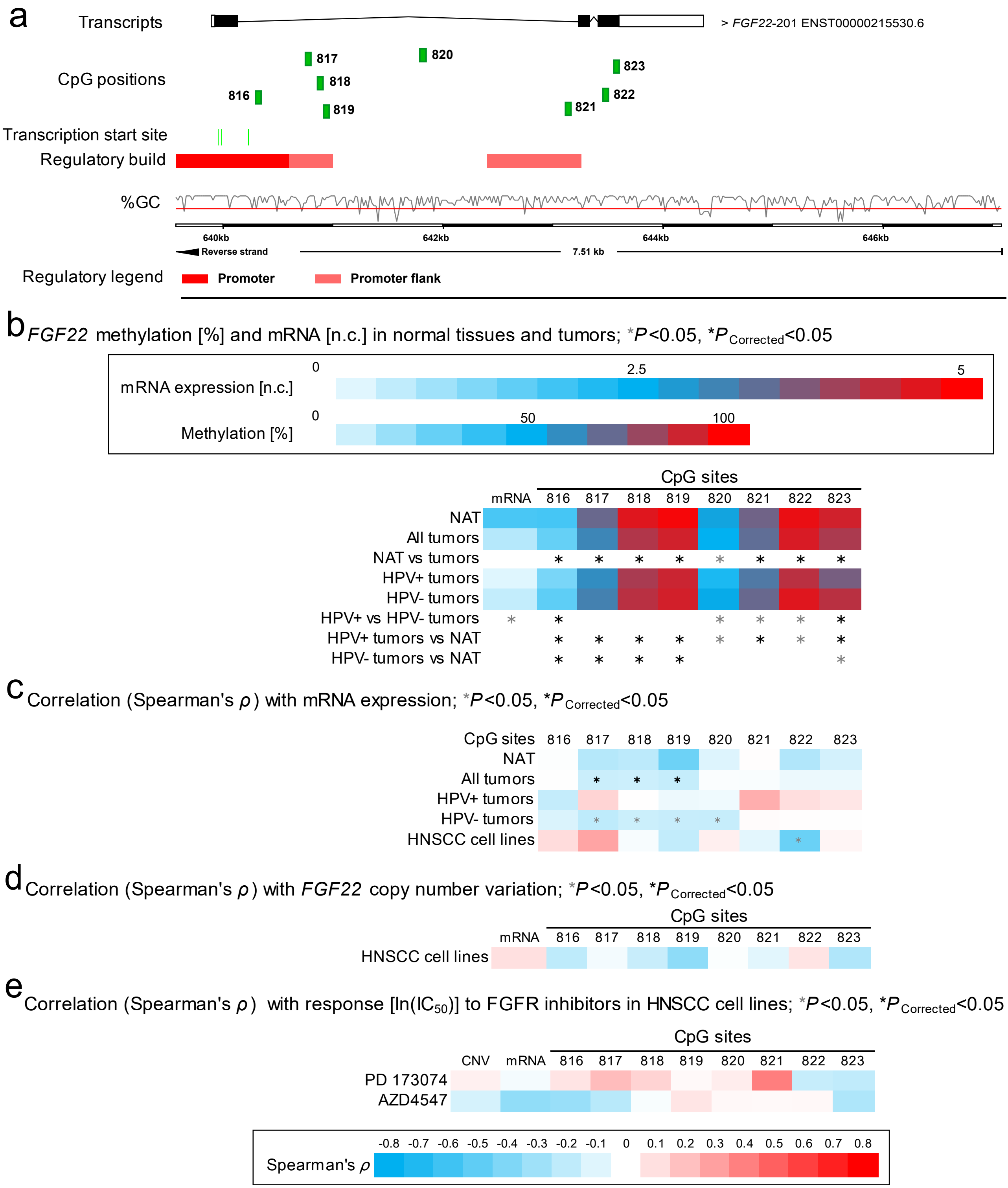
a
Transcripts
> FGF22-201 ENST00000215530.6
CpG positions
816
817
818
819
820
821
822
823
Transcription start site
Regulatory build
%GC
640kb
642kb
644kb
646kb
Reverse strand
7.51 kb
Regulatory legend
Promoter
Promoter flank
b FGF22 methylation [%] and mRNA [n.c.] in normal tissues and tumors; *P<0.05, *P_Corrected<0.05
mRNA expression [n.c.]
0
2.5
5
Methylation [%]
0
50
100
CpG sites
mRNA
816 817 818 819 820 821 822 823
NAT
All tumors
NAT vs tumors
HPV+ tumors
HPV- tumors
HPV+ vs HPV- tumors
HPV+ tumors vs NAT
HPV- tumors vs NAT
c Correlation (Spearman's ρ) with mRNA expression; *P<0.05, *P_Corrected<0.05
CpG sites
816 817 818 819 820 821 822 823
NAT
All tumors
HPV+ tumors
HPV- tumors
HNSCC cell lines
d Correlation (Spearman's ρ) with FGF22 copy number variation; *P<0.05, *P_Corrected<0.05
CpG sites
mRNA
816 817 818 819 820 821 822 823
HNSCC cell lines
e Correlation (Spearman's ρ) with response [ln(IC50)] to FGFR inhibitors in HNSCC cell lines; *P<0.05, *P_Corrected<0.05
CpG sites
CNV
mRNA
816 817 818 819 820 821 822 823
PD 173074
AZD4547
Spearman's ρ
-0.8 -0.7 -0.6 -0.5 -0.4 -0.3 -0.2 -0.1 0 0.1 0.2 0.3 0.4 0.5 0.6 0.7 0.8
