# Supplementary material for: Fibroblast growth factor (FGF), FGF receptor (FGFR), and cyclin D1 (CCND1) DNA methylation in head and neck squamous cell carcinomas is associated with transcriptional activity, gene amplification, human papillomavirus (HPV) status, and sensitivity to tyrosine kinase inhibitors
Source: Clin Epigenetics. 2021 Dec 21;13:228. doi: 10.1186/s13148-021-01212-4 (PMC8693503; doi:10.1186/s13148-021-01212-4)
Supplement: Supplementary file 19 — Additional file 19: Fig. S17. This figure illustrates correlation and association of FGF20 DNA methylation with mRNA expression, HPV status, copy number variation, and sensitivity to the FGFR-targeted TKIs PD 173074 and AZD4547. Exemplarily, results of three selected CpG sites within FGF20 are illustrated. [file 13148_2021_1212_MOESM19_ESM.pdf]

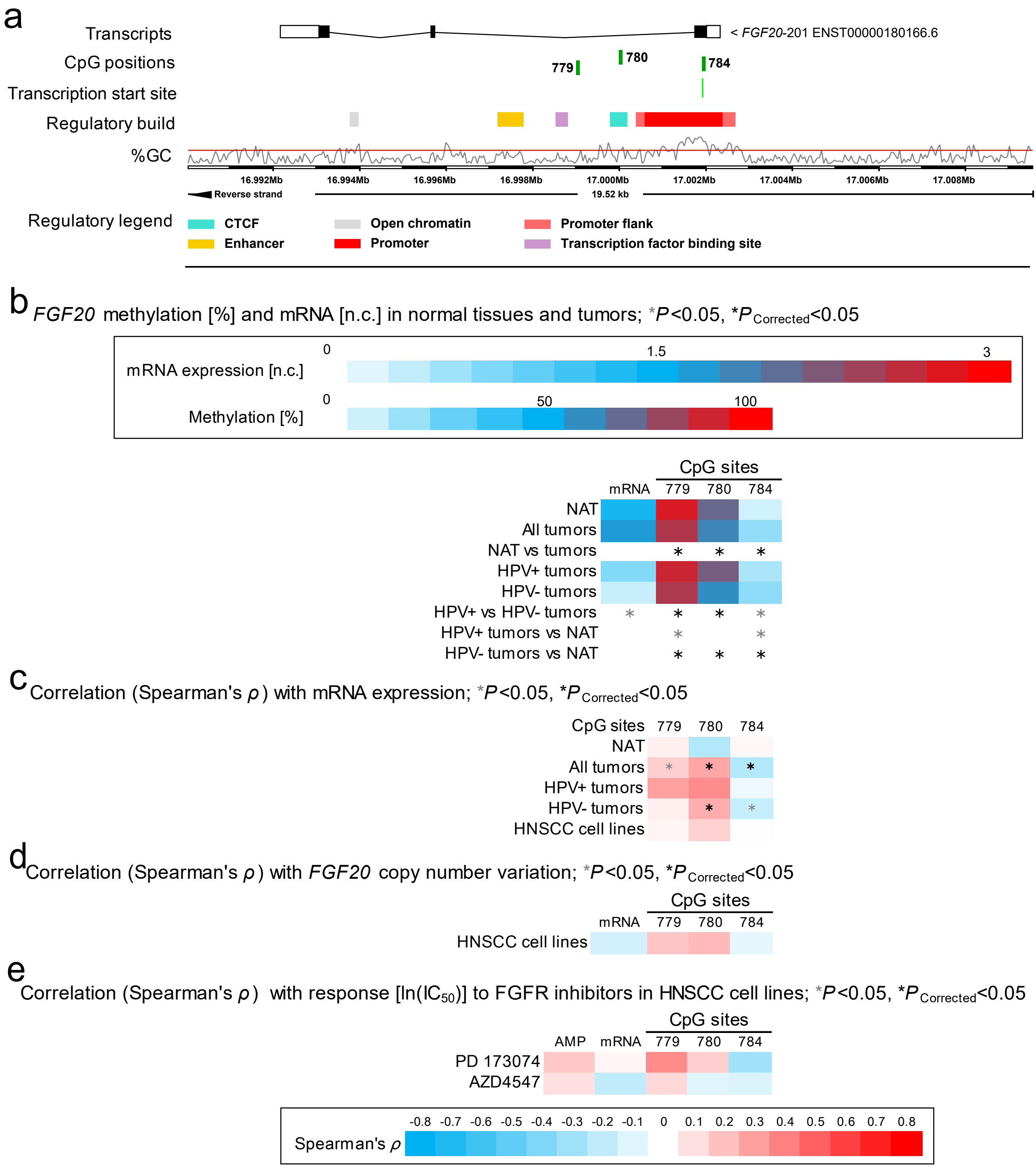
a
Transcripts
< FGF20-201 ENST00000180166.6
CpG positions
779
780
784
Transcription start site
Regulatory build
%GC
16.992Mb
16.994Mb
16.996Mb
16.998Mb
17.000Mb
17.002Mb
17.004Mb
17.006Mb
17.008Mb
Reverse strand
19.52 kb
Regulatory legend
CTCF
Enhancer
Open chromatin
Promoter
Promoter flank
Transcription factor binding site
b
FGF20 methylation [%] and mRNA [n.c.] in normal tissues and tumors; *P<0.05, *P Corrected<0.05
mRNA expression [n.c.]
0
1.5
3
Methylation [%]
0
50
100
CpG sites
mRNA
779
780
784
NAT
All tumors
NAT vs tumors
HPV+ tumors
HPV- tumors
HPV+ vs HPV- tumors
HPV+ tumors vs NAT
HPV- tumors vs NAT
c
Correlation (Spearman's ρ) with mRNA expression; *P<0.05, *P Corrected<0.05
CpG sites
779
780
784
NAT
All tumors
HPV+ tumors
HPV- tumors
HNSCC cell lines
d
Correlation (Spearman's ρ) with FGF20 copy number variation; *P<0.05, *P Corrected<0.05
CpG sites
mRNA
779
780
784
HNSCC cell lines
e
Correlation (Spearman's ρ) with response [ln(IC50)] to FGFR inhibitors in HNSCC cell lines; *P<0.05, *P Corrected<0.05
CpG sites
AMP
mRNA
779
780
784
PD 173074
AZD4547
Spearman's ρ
-0.8
-0.7
-0.6
-0.5
-0.4
-0.3
-0.2
-0.1
0
0.1
0.2
0.3
0.4
0.5
0.6
0.7
0.8
